# Single-cell analysis highlights the significance of malignant cell IFN/MHC-II for immunotherapy response in head and neck squamous cell carcinoma

**DOI:** 10.1016/j.xcrm.2026.102715

**Published:** 2026-03-31

**Authors:** Michael Mints, Reilly A. Sample, Anuraag S. Parikh, Jesse M. Zaretsky, Zongtai Qi, Travis Law, Fudong Wang, Thomas F. Barrett, Riley Mullins, Ashley Reeb, Alissa C. Greenwald, Emily Stoller, Salma Ramadan, Sophie Gerndt, Peter Oppelt, Jessica Ley, Wade Thorstad, Randal C. Paniello, Jason T. Rich, Richard A. Harbison, Paul A. Zolkind, Ryan S. Jackson, Patrik Pipkorn, Douglas R. Adkins, Ravindra Uppaluri, Itay Tirosh, Sidharth V. Puram

**Affiliations:** 1 Department of Molecular Cell Biology, Weizmann Institute of Science, Rehovot, Israel; 2 Department of Oncology-Pathology, Karolinska Institute, Stockholm, Sweden; 3 Department of Otolaryngology-Head and Neck Surgery, Washington University School of Medicine, St. Louis, MO, USA; 4 Department of Genetics, Washington University School of Medicine, St. Louis, MO, USA; 5 Department of Otolaryngology-Head and Neck Surgery, Columbia University Irving Medical Center, New York, NY, USA; 6 Department of Medicine/Medical Oncology, Washington University School of Medicine, St. Louis, MO, USA; 7 The Robert Ebert and Greg Stubblefield Head and Neck Tumor Center at Siteman Cancer Center at Siteman, Washington University School of Medicine, St. Louis, MO, USA; 8 University of Massachusetts Chan Medical School, Worcester, MA, USA; 9 Department of Radiation Oncology, Washington University School of Medicine, St. Louis, MO, USA; 10 Department of Medical Oncology, Dana-Farber Cancer Institute, Boston, MA, USA; 11 Harvard Medical School, Boston, MA, USA; 12 Department of Surgery/Otolaryngology, Brigham and Women’s Hospital, Boston, MA, USA

**Keywords:** single cell RNA-seq, immunotherapy, pembrolizumab, MHC-II, deconvolution, malignant cell biomarker

## Abstract

In head and neck squamous cell carcinoma (HNSCC), immunotherapy response rates remain modest, with difficulty predicting responders. Previous studies characterizing immunotherapy-associated cellular changes in HNSCC focus on immune cells, providing limited insight into malignant cell responses. Here, we perform single-cell RNA sequencing (RNA-seq) on 16 HNSCC patients pre- and post-neoadjuvant pembrolizumab treatment. We identify an interferon (IFN)/major histocompatibility complex class II (MHC-II) expression program in malignant cells, characterized by MHC-II and IFN-response genes, which is associated with response to pembrolizumab. We validate malignant cell MHC-II expression at the protein level via multiplexed immunofluorescence. In a murine HNSCC model, IFN-γ-induced malignant cell MHC-II expression marks immunotherapy-sensitive tumors with favorable immune microenvironments. Finally, we confirm that pre-treatment malignant-IFN/MHC-II is a marker of response through deconvolution of bulk RNA-seq data from an independent cohort. Beyond identifying the malignant IFN/MHC-II program as a potential biomarker for immunotherapy response in HNSCC, our work elucidates the important role of malignant cells in immunotherapy.

## Highlights

• Single cell RNA-seq shows the expression changes induced by pembrolizumab in HNSCC
• A malignant cell subset expresses interferon-response and MHC-II genes (IFN/MHC-II)
• Anti-tumor IFN-γ signaling in malignant cells drives IFN/MHC-II expression
• Malignant IFN/MHC-II associates with favorable immunity and therapeutic response

## Graphical abstract

**Figure:**
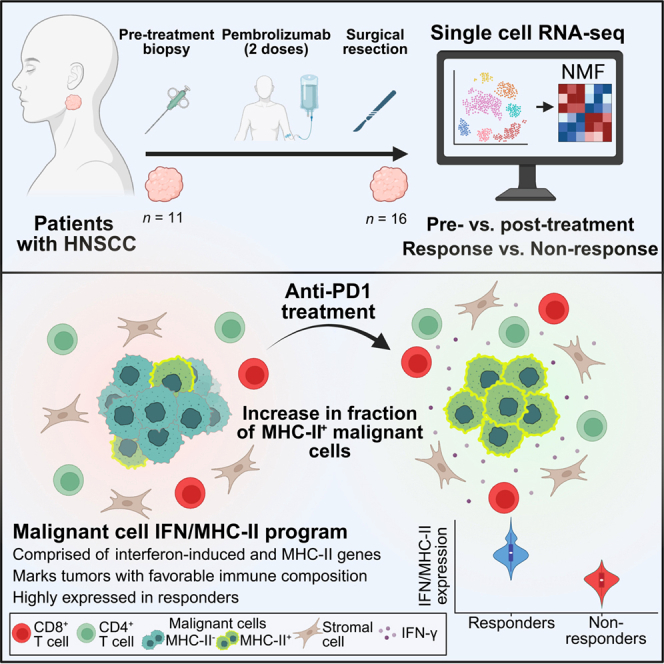

## Teaser

Mints et al. use single cell RNA-seq to show that pembrolizumab reshapes cellular gene expression in patients with HNSCC. A subset of malignant cells expresses interferon-response and MHC-II genes (IFN/MHC-II), driven by anti-tumor interferon-γ signaling. This subset marks tumors with favorable immune microenvironments and response to immunotherapy.

## Introduction

Immunotherapy has emerged as a new treatment modality across oncology, including head and neck squamous cell carcinoma (HNSCC).^1^ In HNSCC, immune checkpoint inhibitors targeting the programmed cell death protein 1 (PD-1)/programmed death-ligand 1 (PD-L1) checkpoint, most notably pembrolizumab and nivolumab, were initially approved only for platinum-refractory HNSCC and later in the metastatic or recurrent/unresectable settings.^1^ Most recently, however, the KEYNOTE-689 phase 3 trial tested perioperative pembrolizumab in resectable HNSCC and demonstrated an 11%–14% event-free survival benefit,^2^ establishing a new standard of care in HNSCC.

Still, response rates in recurrent/metastatic HNSCC remain modest, averaging 15%–20%.^3^^,^^4^ This limited efficacy underscores the need to identify responders *a priori*, allocating predicted responders to immunotherapy and directing others toward alternative approaches. Current biomarkers in use include the tumor proportion score (TPS), a measure of PD-L1 positivity among tumor cells; tumor-infiltrating immune cells (IC), a measure of PD-L1 positivity among the immune infiltrate; and the combined positive score (CPS), a combination of TPS and IC.^4^^,^^5^ However, in the KEYNOTE-012 trial, TPS did not predict response rate, and while higher response rates were observed in patients with positive PD-L1 expression by CPS, more than 80% of tumors were positive, yet the overall response rate in this group was only 21%.^6^ Collectively, these results highlight the need for better biomarkers.

Many studies have characterized the cells associated with neoadjuvant immunotherapy response in HNSCC,^7^^,^^8^^,^^9^^,^^10^^,^^11^^,^^12^ with most focused on immune cells. In the pre-treatment setting, Oliveira et al. reported that response to neoadjuvant pembrolizumab correlated with high numbers of infiltrating *CD103+PD-1+CD8*^*+*^ T cells.^7^ Similarly, other studies identified subpopulations of blood *PD-1+/KLRG1(−)* T cells^8^ and tumor-infiltrating *4-1BB+/OX-40+* active Tregs^9^ associated with response to PD-1 and CTLA-4 blockade prior to therapy. Post-treatment, immunotherapy response has been associated with increased tissue-resident memory and cytotoxicity programs in CD8^+^ T cells,^8^ decreased CD8^+^ T cell dysfunction,^9^ reduced intra-tumoral retention of exhausted CD8^+^ T cells,^10^ and CD4^+^ T cell activation and recruitment from lymph nodes.^11^

Although these studies established an understanding of T cell responses to immunotherapy, their focus on the immune compartment has left open questions about how malignant cells respond to treatment and contribute to therapeutic sensitivity or resistance. Motivated by this gap, we performed single-cell RNA sequencing (scRNA-seq) on HNSCC patients pre- and post-neoadjuvant pembrolizumab treatment, profiling 137,020 cells from 16 patients. We uncovered the landscape of cellular expression changes upon immunotherapy treatment, not only among immune cells but also among malignant and stromal cells. Strikingly, we identified an interferon (IFN)/ major histocompatibility complex class II (MHC-II) program in malignant cells, characterized by expression of MHC-II and IFN-response genes, which was associated with tumor response in both patient samples and syngeneic murine models of HNSCC. These findings highlight the importance of malignant cell states for immunotherapy response and suggest that the malignant-IFN/MHC-II program may be of interest as a biomarker of response in HNSCC.

## Results

### Profiling the HNSCC tumor ecosystem in patients undergoing immunotherapy

**Figure 1:**
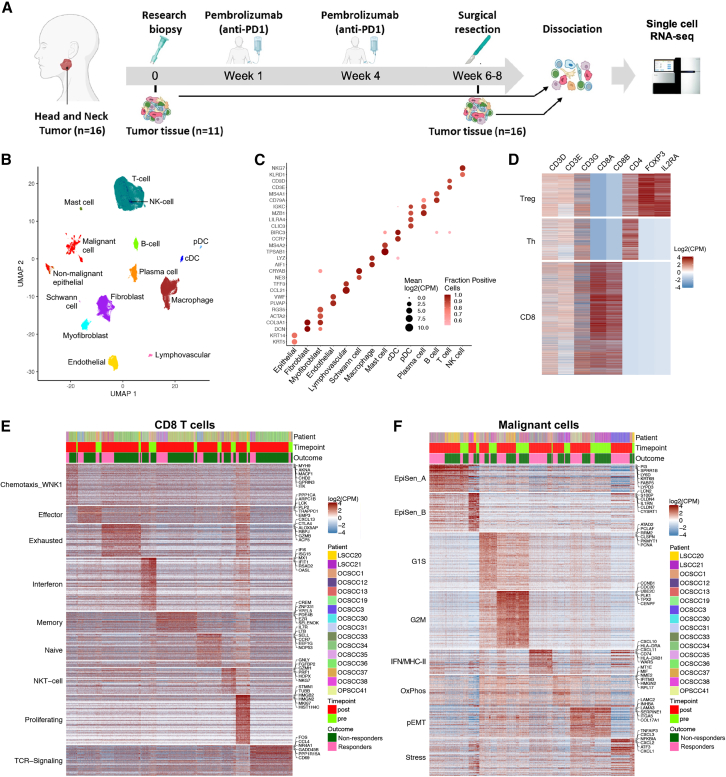
Single-cell RNA-seq in pre- and post-immunotherapy HNSCC samples (A) Schematic shows workflow of sample collection and sequencing. (B) Uniform manifold approximation and projection shows 117,012 cells that passed QC and had a confidently assigned cell type colored by cell type. (C) Dot plot shows expression of selected marker genes (*y* axis) by all cells assigned to each cell type (*x* axis). Dot size represents average expression, and dot color represents the fraction of cells with non-zero expression. (D) Heatmap shows 30,377 T cells that could be confidently assigned into CD8, T helper (Th), and T regulatory (Treg) subsets. (E) Heatmap shows expression of meta-program genes (rows) in all CD8 T cells (columns). Cells are annotated by patient of origin, sampling time point, and outcome. (F) Heatmap shows expression of meta-program genes (rows) in all malignant cells (columns). Cells are annotated by patient of origin, sampling time point, and outcome.

To comprehensively evaluate the transcriptional landscape of HNSCC tumors in the context of immunotherapy, we used scRNA-seq to profile matched HNSCC patient samples before (*n* = 11) and after (*n* = 16) treatment with two cycles of the anti-PD-1 agent pembrolizumab (Figure 1A). Patients had stage III/IV HNSCC and were part of cohort 2 of a multicenter phase 2 clinical trial, with outcomes and pathologic response previously reported.^7^ Notably, results from this study formed the basis for the recent phase 3, randomized KEYNOTE-689 trial. Matched pre- and post-treatment samples were available for 10 patients, including five responders and five non-responders. Pathologic response was defined as percent area of tumor regression, with responders classified as pTR-2 (>50% tumor regression) or pTR-1 (10%–50%), while <10% tumor regression was considered non-response. Overall, we profiled 179,560 cells and analyzed 137,020 cells that passed QC. Based on gene expression, clustering, and expression of specific marker genes, cells were assigned to 15 distinct cell types, including epithelial cells, immune cell types (T cells, B cells, macrophages, plasma cells, mast cells, and conventional and plasmacytoid dendritic cells), and stromal cell types (fibroblasts, myofibroblasts, endothelial cells, lymphovascular cells, and Schwann cells) (Figures 1B and 1C).

As malignant and non-malignant epithelial cells shared a similar transcriptional signature, we separated epithelial cells into malignant and non-malignant subsets by inferring copy-number aberrations (CNAs), as previously done by our group.^13^^,^^14^^,^^15^ Briefly, a 100-gene moving average was applied to all genes and compared to stromal cells from the same patient to identify changes in gene expression impacting a large number of genes grouped together on a chromosome, allowing us to infer CNAs and genetic subclones (Figure S1A, representative tumor). Then, by comparing the absolute value of each epithelial cell across regions with CNAs (CNA signal), as well as the cell’s similarity to other cells with CNAs (CNA correlation), epithelial cells were assigned as malignant, non-malignant, or unresolved (Figure S1B; STAR Methods).

Since the various T and natural killer (NK) cell subsets tended to cluster together based on their transcriptional similarity, we performed a more stringent subdivision into T cell subsets. We first subdivided cells based on expression of canonical markers, then performed differential gene expression between the canonical subsets, and finally used recurrent, differentially expressed genes to score the remaining cells lacking canonical markers and assign them to a subset. We applied this approach in the following order: (1) T cells vs. NK cells based on the presence of T cell receptor (TCR), (2) CD8 vs. CD4 T cells based on the presence of *CD8A/CD8B* or CD4, and (3) CD4 T regulatory vs. T helper cells based on the presence of *FOXP3* and *IL2RA* (Figure 1D; STAR Methods). Clustering of cells by patient was also performed (Figure S1C).

After assigning cell types, we examined within-type heterogeneity to identify cellular states and their relationships to treatment and outcome. We applied non-negative matrix factorization (NMF), previously used by our group across many datasets,^13^^,^^14^^,^^15^^,^^16^^,^^17^ to uncover programs of cellular states that recurred across multiple patients. While common clustering approaches can uncover discrete cellular subpopulations, these approaches are skewed by sample-intrinsic properties and are less adept at finding continuous patterns of intratumor heterogeneity. NMF, applied to each individual cell type and patient sample, decomposes the gene-cell expression matrix into additive gene expression programs, uncovering sample-specific patterns of transcriptional heterogeneity per cell type. Next, by clustering these sample-specific gene expression programs (based on their number of overlapping genes) and extracting the recurrent genes from each cluster of programs, we defined metaprograms, reflecting robust, recurrent patterns of intra-tumoral heterogeneity. This approach enabled us to describe the heterogeneity within each cell type at high resolution (Table S1; STAR Methods).

Analysis of CD8^+^ T cells identified 9 cellular states, each characterized by dozens of genes (Figures 1E and S1D). These features are in line with differentially expressed genes across high-resolution T cell subclusters from other published scRNA-seq atlases of tumor-infiltrating lymphocytes.^18^^,^^19^ Some of these expression programs were associated with distinct phenotypes, such as proliferating (*MKI67*), exhausted (*CXCL13*, *CTLA4*, *ENTPD1*, and *LAG3*), or NK-like (*GNLY*, *FGFBP2*) subsets. Other programs represented additive activity states. For example, within memory cells (*GZMK* and *CREM*), we observed variable contributions from programs of naive-like (*IL7R*, *LTB*, *SELL*, and *CCR7*) and TCR signaling (*FOS*, *JUN*, *NR4A1*, *TNF*, and *IFNG*^20^). Additional activity states were broadly distributed, relating to effector function (*GZMA* and *GZMH*), IFN response (*IFIT1* and *ISG15*), and chemotaxis (*WNK1*).^21^

Similarly, we analyzed the diversity among malignant cells and identified the main cellular states found previously in HNSCC. These included a hybrid, partial epithelial-mesenchymal transition program (p-EMT)^13^ represented by genes such as *LAMC2* and *INHBA* and two related but distinct states associated with epithelial senescence (EpiSen) and defined by genes such as *S100A8/A9*, *SPRR1B*, and *CDKN1A*.^13^^,^^14^ EpiSen_A represented a more differentiated epithelial state, expressing multiple keratins (*KRT6*/*16*/*17*), whereas EpiSen_B uniquely expressed *LCN2*, *CLDN4*, and *CLDN7* (Figures 1F and S1E). Notably, one malignant state, which we denoted IFN/MHC-II, was associated *primarily* with MHC-II (*HLA-DR*, *HLA-DP*, *HLA-DQ*, and *CD74*) and IFN-response genes (*CXCL9*/*10*/*11*, *TAP1*, *IFIT1*, and *IRF1*). In total, 7/37 genes in this program were MHC-II genes and 18/37 were IFN-response genes (Table S1), motivating the naming and suggesting a potential interplay with immune cell types.

Similar analysis was also performed for T helper cells (Figures S1F–S1G),^18^^,^^22^ T regulatory cells (Figures S1H and S1I),^23^^,^^24^^,^^25^ and six other cell types with adequate cell numbers for analysis of recurrent cell states (Figure S2). Interestingly, beyond the malignant and immune cells, we also found states associated with IFN-response genes in stromal populations including endothelial cells (Figure S2B), fibroblasts (Figure S2C), and myofibroblasts (Figure S2E). These programs may reflect a potential crosstalk with immune cells that is not cell-type specific, such as response to high levels of IFN-γ produced by T cells.

### T cell state composition shifts following anti-PD1 treatment

We next compared the patterns of T cell subsets before and after pembrolizumab treatment. After pembrolizumab, the fraction of CD8^+^ T cells increased and the fraction of CD4^+^ T cells decreased significantly in 8 of 9 patients (Figure S3A). In contrast, the fraction of Tregs did not change in a consistent manner across patients.

**Figure 2:**
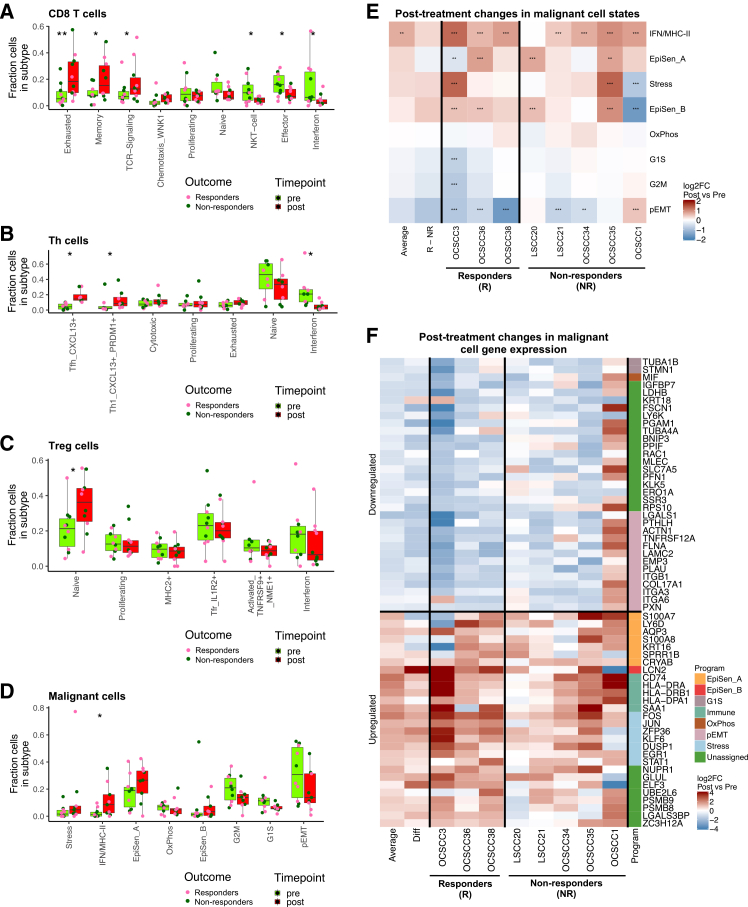
T cell and malignant cell subsets are altered following anti-PD1 treatment highlighted by post-treatment upregulation of malignant cell MHC-II and interferon-response genes. (A–D) For each meta-program, boxplots and points show the fraction of CD8 T (A), T helper (B), T regulatory (C), and malignant (D) cells, per patient and time point, assigned to that meta-program, before (light green) and after (red) treatment. Points are colored by responders (pink) and non-responders (dark green). Asterisks (∗*p* < 0.05, ∗∗*p* < 0.01, ∗∗∗*p* < 0.001) denote that metaprogram proportions are significantly changed after treatment (paired *t* test). Only patients with at least 20 cells confidently assigned to a metaprogram both pre- and post-treatment were kept for analysis. (E) Heatmap shows differences in malignant metaprogram scores for each single patient with at least 50 cells at each time point. The average is derived by averaging all mean values, the R − NR column is the difference between responder and non-responder means. Asterisks denote significance by *t* test (∗*p* < 0.05, ∗∗*p* < 0.01, ∗∗∗*p* < 0.001). Only fold changes above 1.3 were marked as significant. (F) For each patient with at least 50 malignant cells at both time points, heatmap shows change in gene expression after treatment for all genes significantly (*p* < 0.05 and log2(FC)≥1) up/downregulated in at least half of all patients. Genes are annotated by metaprogram assignment. The average is derived by averaging all mean values; the Diff column is the difference between responder and non-responder means.

In each of these three T cell subsets, we further identified an effect of anti-PD1 treatment on the relative proportions of distinct cellular states. In CD8^+^ T cells, we found increased proportions (*p* < 0.05, paired *t* test) of exhausted, memory, and TCR signaling programs and decreased proportions of IFN, effector, and NK-like states post-treatment (Figures 2A and S3B). In CD4^+^ T helper cells, we found increased proportions of T follicular helper and Th1-like states^18^^,^^22^ and decreased proportions of the IFN-response state (Figure 2B). Finally, in Tregs, we found an increased proportion of the naive state (Figure 2C), perhaps reflecting a reciprocal decrease in more activated states. Interestingly, in both CD8 and T helper cells, the IFN state was depleted after treatment (Figures 2A and 2B).

### Malignant cells upregulate MHC-II and downregulate p-EMT following anti-PD1 treatment

We next focused on changes in malignant and stromal cell types upon anti-PD1 treatment, which have largely been excluded in prior studies.^7^^,^^8^ Malignant cells showed treatment-associated genetic and expression changes. In eight of 11 patients, genetic subclones were significantly linked to sampling time point (*p* < 0.05, chi-square test) (Figure S3C), including one exceptional patient with completely unique subclones at the pre- and post-treatment time points (Figure S3D). Although these genetic differences may reflect sampling variation pre- vs. post-treatment, the consistent observation of genetic changes across time points suggests enrichment or selection of specific genetic subclones following anti-PD1 treatment.

We next studied how malignant cell expression states changed upon pembrolizumab treatment. We noted a significant increase in the proportion of malignant cells exhibiting the IFN/MHC-II state after treatment (*p* < 0.05, *t* test). By contrast, the fraction of cells exhibiting the p-EMT state trended toward post-treatment depletion (*p* = 0.07, *t* test) (Figure 2D). We also compared the cell state signature scores for each sample, which account for the continuous nature of cell states and the fact that one cell may express genes from multiple signatures simultaneously. Here, we similarly noted a consistent increase in the IFN/MHC-II signature score across 7 of 8 patients. By contrast, a significant decrease in p-EMT was seen in a subset of patients, including all responders but only two of the five non-responders (Figure 2E).

We then performed differential gene expression analysis between malignant cell populations pre- and post-treatment. For each patient with samples from both time points, we defined genes significantly changed (*p* < 0.05, paired *t* test and absolute log2FC > 1). We then identified genes that were consistently affected in at least 4 of 8 patients, without limiting the analysis to genes from the NMF-defined cell state signatures (Table S2). Several MHC-II genes (*HLA-DRA*, *HLA-DRB1*, *HLA-DPA1*, and *CD74*) were among the consistently upregulated genes in post-treatment samples (Figures 2F and S3E), as were multiple stress response genes (*FOS*, *JUN*, and *EGR1*) and genes of the EpiSen programs (*S100A8*, *SPRR1B*, and *AQP3*). These four MHC-II genes were among the 11 genes that were upregulated in at least 5/8 patients (Figure 2F), highlighting the consistency of post-treatment MHC-II upregulation. On the other hand, p-EMT-related genes such as *LAMC2*, *PLAU*, *COL17A1*, and multiple integrins were consistently downregulated across samples (except for OCSCC1), supporting the trends found when analyzing cell state composition and signatures (Figure 2F).

### Malignant cell MHC-II is upregulated after pembrolizumab treatment at the protein level

**Figure 3:**
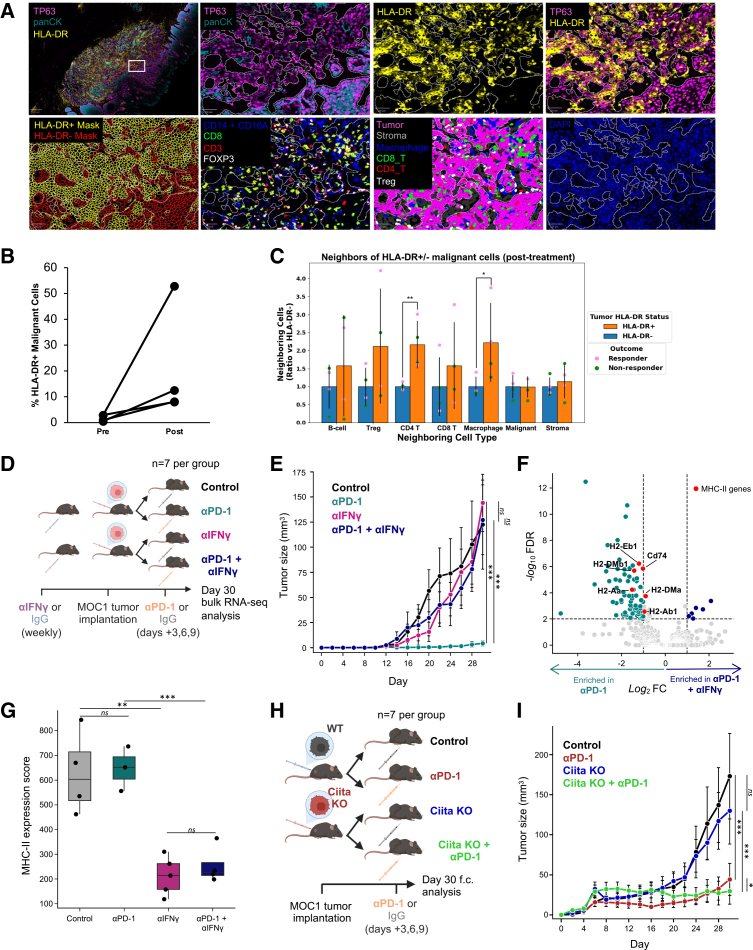
Malignant cell-specific expression of MHC-II is IFN-γ dependent but dispensable for anti-PD1-mediated tumor control (A) Post-treatment multiplex immunofluorescence (mIF) PhenoCycler images of formalin-fixed paraffin-embedded-stained tissues show representative areas of high HLA-DR expression on malignant cells from a representative tumor (OCSCC3). Top left image shows whole-slide view, while other panels represent magnified fields of view from inset, showing (from top left to bottom right) malignant cells highlighted by TP63 and panCK, HLA-DR expression, HLA-DR expression within malignant cells, assigned cell boundaries following Instanseg cell segmentation and HLA-DR +/− classification based on centered log-ratio threshold, staining for T cell and macrophages (CD14 and CD16A combined in blue, CD8 in green, CD3 in red, and FOXP3 in white), post-classification cell-type assignment, and DNA stain for all cells. (B) Line plots show percent of HLA-DR-positive malignant cells by mIF, pre- and post-treatment. Lines connect paired samples from individual patients (*n* = 4). (C) Bar plots show relative count of seven neighboring cell types within a 30 μm radius of malignant cells that are HLA-DR+ (orange bars) and HLA-DR− (blue bars). Bars show counts relative to values for HLA-DR- malignant cells. Pink dots represent responders, while green dots represent non-responders. Error bars represent the SEM. Asterisks (∗*p* < 0.05, ∗∗*p* < 0.01) denote significance by unpaired one-sided *t* test, HLA-DR+ greater than HLA-DR−. HLA-DR+ malignant cells are surrounded by greater numbers of CD4 T cells (*p* = 0.006) and macrophages (*p* = 0.036) than HLA-DR− malignant cells. (D and E) (D) Schematic for (E–G). C57BL/6 mice received anti-IFN-γ or IgG control 1 week prior to tumor implantation and once weekly starting at tumor implantation. MOC1 tumor-bearing mice then received anti-PD1 or IgG control antibodies on days 3, 6, and 9 post-tumor implantation. Growth of tumors was tracked until day 30 post-implantation. Tumors were then harvested for bulk RNA-seq. (E) Growth of tumors from (D). Error bars denote standard error between mice within each treatment group. Asterisks (∗∗∗*p* < 0.001) denote significance by *t* test at day 30. (F) Volcano plot shows genes with largest expression differences between tumor-bearing mice treated with anti-PD1 vs. anti-PD1 + anti-IFN-γ. Red dots highlight genes involved in MHC-II presentation. (G) Boxplot shows MHC-II expression score by bulk RNA-seq of MOC1 tumors from (D). Each dot represents one mouse. Asterisks (∗∗*p* < 0.01, ∗∗∗*p* < 0.001) denote significance by *t* test. (H and I) (H) Schematic for (I). WT or *Ciita* KO MOC1 cell lines were implanted into C57BL/6 mice. Tumor-bearing mice received anti-PD1 or IgG control antibodies on days 3, 6, and 9 post-tumor implantation. Growth of tumors was tracked until day 30 post-implantation. Tumors were then harvested for flow cytometry analysis. (I) Growth of tumors from (H). Error bars denote standard error between mice within each treatment group. Asterisks (∗*p* < 0.05, ∗∗∗*p* < 0.001) denote significance by *t* test.

Having observed MHC-II transcriptional upregulation in malignant cells post-treatment, we sought to validate this finding at the protein level and explore spatial correlations with immune cells. We performed cyclic multiplexed immunofluorescence using Co-Detection by Indexing (CODEX) with a custom panel of 29 markers to capture malignant, immune, and stromal cell types (STAR Methods). CODEX was applied to eight representative samples matched to our scRNA-seq data, including two responders and two non-responders, each with paired pre- and post-treatment time points. After cell segmentation and phenotyping (Figures 3A, S3F, and S3G), we observed clear membranous HLA-DR expression on malignant cells (Figure 3A), consistent with true malignant cell-intrinsic MHC-II expression rather than spillover from immune cells, as HLA-DR-negative malignant cells were also observed adjacent to (and demarcated from) HLA-DR-positive immune cells (Figure S3F). The fraction of malignant cells expressing HLA-DR increased post-treatment in all samples (Figure 3B), consistent with treatment-induced changes in the tumor microenvironment—potentially driven by IFN signaling—promoting MHC-II expression on malignant cells. Finally, we assessed cellular spatial relationships of HLA-DR+ and HLA-DR− malignant cells and found that HLA-DR+ malignant cells had more nearby macrophages and CD4^+^ T cells than HLA-DR− malignant cells post-treatment (Figure 3C, unpaired one-sided *t* test, *p* < 0.05).

### Malignant cell-specific MHC-II expression is IFN-γ dependent but dispensable for tumor control under PD-1 blockade

Given the proximity of HLA-DR+ malignant cells to inflammatory cells, we next sought to understand the regulation and functional importance of malignant cell MHC-II expression. To do so, we leveraged the MOC1 cell line model of HNSCC,^26^ a C57BL/6 syngeneic 7,12-dimethylbenz(a) anthracene (DMBA)-induced mouse oral squamous cell carcinoma known to be largely responsive to anti-PD1 immunotherapy. While some studies have shown IFN-γ to regulate MHC-II expression,^27^^,^^28^^,^^29^^,^^30^ the degree to which malignant cell-specific MHC-II induction relies on IFN-γ *in vivo* remains unknown. To test whether IFN-γ was required for MHC-II upregulation and PD-1 response in this model, we administered an IFN-γ-neutralizing antibody or an isotype IgG control to C57BL/6 mice beginning 1 week before implantation of MOC1 tumors, and continuously every 7 days throughout the duration of the experiment. Mice were also treated with anti-PD-1 or control IgG on days 3, 6, and 9 post-tumor implantation (Figure 3D).

MOC1 tumors receiving anti-PD-1 therapy alone responded to treatment, while tumors treated with anti-PD-1 plus anti-IFN-γ failed to respond (Figure 3E). These findings confirm that IFN-γ signaling is critical for the antitumor effects of PD-1 blockade. We subsequently examined the global gene expression changes in malignant cells associated with IFN-γ blockade. We performed bulk RNA barcoding and sequencing (BRB-seq) on MOC1 tumors from mice treated with anti-PD-1 with or without IFN-γ blockade, as well as their respective controls. Consistent with our hypothesis, MHC-II genes (e.g., *H2-Eb1*, *Cd74*, *H2-DMb1*, and *H2-Ab1*) were among the top downregulated genes after anti-IFN-γ administration in anti-PD-1-treated mice (Figure 3F; Table S3). Interestingly, we observed a marked decrease in MHC-II expression scores in all tumors that received the IFN-γ-neutralizing antibody (Figure 3G). This reduction was not rescued by anti-PD-1 therapy, suggesting that IFN-γ is necessary for malignant cell MHC-II expression under PD-1 blockade.

We next assessed whether malignant cell MHC-II was functionally important for anti-PD-1-mediated tumor control. Although some studies have reported that malignant cell-specific MHC-II can directly influence antitumor immunity, others have shown minimal effects depending on the cancer type and context.^31^ To clarify this in our model, we knocked out the master transcriptional regulator of MHC-II, *Ciita*, which is required for IFN-γ-dependent induction of MHC-II^32^ (Figure S4A). Importantly, given *Ciita*’s minor role in MHC-I regulation,^33^ we confirmed that *Ciita* KO does not impair IFN-γ-induced MHC-I expression. We then implanted either wild-type (WT) or *Ciita* KO MOC1 cells into WT C57BL/6 mice and treated them with anti-PD-1 or control IgG on days 3, 6, and 9 post-implantation (Figure 3H). We observed no significant difference in growth between mice bearing WT and *Ciita* KO tumors (Figure 3I). Moreover, loss of malignant cell-specific MHC-II expression did not significantly affect infiltrating immune cell proportions under PD-1 blockade (Figure S4B). Together, these results suggest that while IFN-γ-mediated MHC-II upregulation in malignant cells is a hallmark of productive antitumor response, the presence of MHC-II on malignant cells alone is not required for therapeutic response to PD-1 blockade.

### IFN-γ signaling in malignant cells sustains MHC-II expression and is required for anti-PD-1 therapeutic response

**Figure 4:**
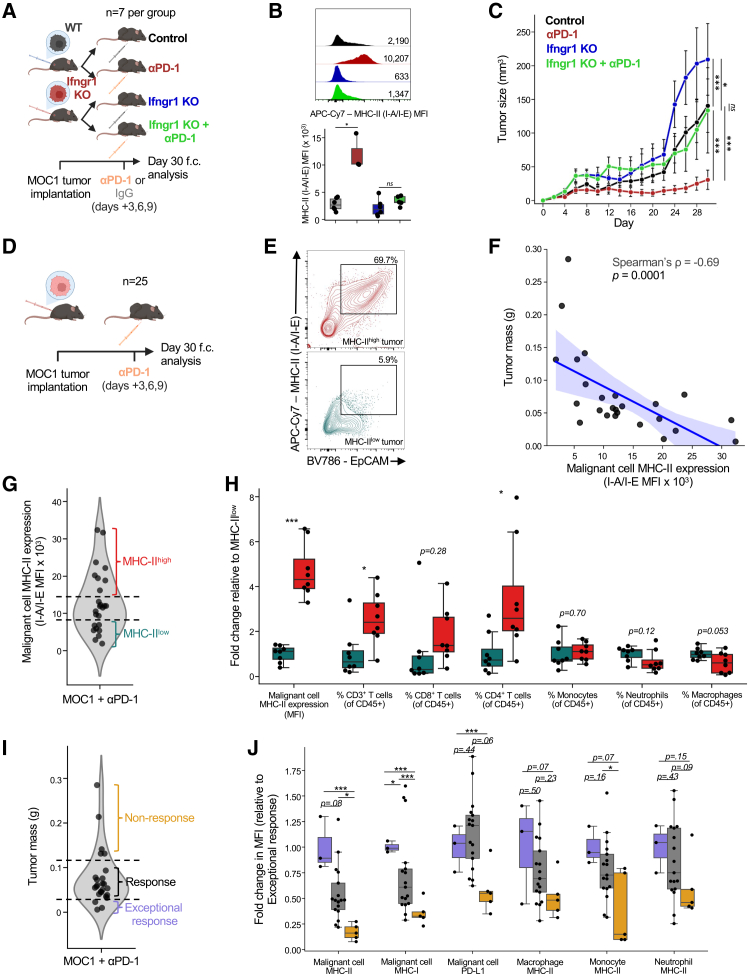
Malignant cell-specific IFN-γ signaling sustains MHC-II expression and correlates with a favorable immune microenvironment and therapeutic response under PD-1 blockade (A) Schematic for (B and C). WT or Ifngr1 KO MOC1 cells were implanted into C57BL/6 mice. Tumor-bearing mice received anti-PD1 or IgG control antibodies on days 3, 6, and 9 post-tumor implantation. Growth of tumors was tracked until day 30 post-implantation. Tumors were then harvested for flow cytometry analysis. (B) (Top) Representative flow cytometry plots for malignant cell-specific MHC-II (I-A/I-E) expression (gated: live cells/CD45^−^/EpCAM^+^/I-A/I-E^+^). Expression was determined by MFI. (Bottom) Boxplot shows malignant cell-specific MHC-II expression for each group from (A). Each dot represents one mouse. Asterisk (∗*p* < 0.05) denotes significance by *t* test. (C) Growth of tumors from (A). Error bars denote standard error between mice within each treatment group. Asterisks (∗*p* < 0.05, ∗∗*p* < 0.01, ∗∗∗*p* < 0.001) denote significance by *t* test. (D) Schematic for (E–J). WT MOC1 tumors were implanted into C57BL/6 mice. Tumor-bearing mice received anti-PD1 or IgG control antibodies on days 3, 6, and 9 post-tumor implantation. Tumors were harvested for flow cytometry analysis on day 30 post-implantation. (E) Representative flow cytometry plots for MHC-II^high^ and MHC-II^low^ tumors from (D). (F) Scatterplot shows, for each mouse from (D), malignant cell-specific MHC-II MFI (*x* axis) and the tumor mass (*y* axis). Spearman correlation and *p* value are shown. Each dot represents one mouse. Data were pooled from 3 independent experiments. (G) Violin plot shows the distribution of MHC-II expression from (D). The top 8 tumors were classified as MHC-II^high^; the bottom 8 tumors were classified as MHC-II^low^. (H) Boxplots show malignant cell-specific MHC-II expression and infiltrating immune cell types between MHC-II^high^ and MHC-II^low^ tumors. Values for each comparison were scaled by the average of MHC-II^low^ tumors. T cells were gated live cells/CD45^+^/CD3^+^. Monocytes were gated live cells/CD45^+^/CD3^−^/CD11b^+^/Ly-6C^+^/Ly-6G^−^. Neutrophils were gated live cells/CD45^+^/CD3^−^/CD11b^+^/Ly-6G^+^/Ly-6C^int^. Macrophages were gated live cells/CD45^+^/CD3^−^/CD11b^+^/Ly-6G^−^/Ly-6C^−^/F4/80^+^. Asterisks (∗*p* < 0.05, ∗∗*p* < 0.01, ∗∗∗*p* < 0.001) denote significance by *t* test. (I) Violin plot shows the distribution of tumor masses from (D). The top 5 tumors were classified as non-response; the middle 17 tumors were classified as response; the bottom 3 tumors were classified as exceptional response. (J) Boxplots show potential response markers (tumor-specific MHC-II, MHC-I, PD-L1, and myeloid MHC-II) across non-responding, responding, and exceptional-response tumors. Values for each comparison were scaled by the average expression in exceptional-response tumors. Asterisks (∗*p* < 0.05, ∗∗∗*p* < 0.001) denote significance by *t* test.

Given that blocking IFN-γ abrogated the efficacy of PD-1 blockade, we next asked whether the IFN-γ signaling that underlies this effect occurs specifically within malignant cells, rather than solely systemically, affecting the priming of CD4^+^ and CD8^+^ T cells by antigen-presenting cells. To ablate malignant cell IFN-γ signaling, we knocked out *Ifngr1*, which encodes the alpha chain of the IFN-γ receptor, thereby preventing assembly of the complete receptor complex (Figure S4C). WT or *Ifngr1* KO MOC1 cells were implanted into mice, which subsequently received anti-PD-1 therapy or IgG control on days 3, 6, and 9 post-tumor implantation (Figure 4A). Strikingly, flow cytometric analysis of malignant cells (defined as CD45^–^EpCAM^+^) revealed a dramatic reduction in malignant cell-specific MHC-II expression among the anti-PD-1-treated *Ifngr1* KO tumors relative to WT (Figure 4B, top). Moreover, MHC-II expression could not be rescued by anti-PD-1 in *Ifngr1* KO tumors (Figure 4B, bottom), suggesting that IFN-γ signaling in malignant cells is required for MHC-II upregulation in the context of PD-1 blockade.

Importantly, loss of malignant cell-intrinsic IFN-γ signaling conferred resistance to PD-1 blockade, with *Ifngr1* KO MOC1 tumors failing to respond to therapy (Figure 4C). Compared to WT tumors, *Ifngr1* KO tumors receiving anti-PD-1 trended toward a lower proportion of T cells (both CD4^+^ and CD8^+^) of all CD45^+^ cells and a higher proportion of macrophages and neutrophils (Figures S4D and S4E). Taken together, these findings suggest that malignant cell-specific IFN-γ signaling may promote a more favorable immune microenvironment and is required for the antitumor efficacy of PD-1 blockade.

### High malignant cell-intrinsic MHC-II expression is a hallmark of ongoing anti-tumor immune responses

To further investigate the relationship between malignant cell-intrinsic MHC-II expression and response to anti-PD1 therapy, we implanted 25 mice with WT MOC1 cells and treated them with anti-PD-1 on days 3, 6, and 9 post-implantation (Figure 4D). Consistent with previous reports, we observed substantial heterogeneity in MOC1 responses to anti-PD-1 therapy, as approximately 20% of tumors developed resistance and progressed, while others underwent partial or complete regression—variability that we leveraged to compare transcriptional differences, including MHC-II expression, among isogeneic tumors with divergent outcomes^34^ (Figures 4E and 4F). Across all anti-PD-1-treated tumors, malignant cell-specific MHC-II expression assessed by flow cytometry exhibited marked variation and was strongly correlated with tumor size: larger, non-responding tumors expressed low malignant cell-specific MHC-II, while smaller, responding tumors exhibited robust MHC-II expression on malignant cells (Figure 4F).

To elucidate immune differences between tumors with high or low malignant cell-specific MHC-II expression, we stratified the top and bottom eight tumors by malignant cell-specific MHC-II mean fluorescence intensity (MFI) (Figure 4G) and compared their proportions of infiltrating immune cells. MHC-II^high^ tumors had significantly higher infiltration of all T cells and CD4^+^ T cells and a trend toward higher infiltration of CD8^+^ T cells. Conversely, MHC-II^low^ tumors had significantly lower proportions of T cells, with a trend toward higher proportions of macrophages and neutrophils (Figure 4H). Thus, malignant cell-specific MHC-II expression appears to be a marker of a favorable immune microenvironment under PD-1 blockade.

Finally, to compare MHC-II expression with other potential markers of therapeutic response, we analyzed malignant cell-specific MHC-I and PD-L1, as well as MHC-II expression on myeloid subsets, in non-responding, responding, and exceptional-responder tumors (defined as those with tumor sizes significantly smaller than the cohort median) (Figure 4I). Among these markers, malignant cell-specific MHC-I and MHC-II best discriminated between the three groups (Figure 4J). While malignant cell-specific PD-L1 performed well in distinguishing non-response from response, PD-L1 expression did not differ significantly between tumors with ongoing exceptional response and response—unlike malignant cell-specific MHC-II (Figure 4J). Notably, MHC-II expression on myeloid cells did not differ significantly between the three groups, further suggesting that malignant cell-specific MHC-II better reflects the anti-tumor IFN-γ signaling activity underpinning anti-PD-1 response (Figure 4J). Thus, our work highlights malignant cell-intrinsic MHC-II expression as a robust indicator of IFN-γ-driven anti-tumor immunity following anti-PD-1 therapy and a potential marker of response.

### Pre-treatment malignant cell-specific MHC-II expression correlates with response to anti-PD1 therapy

**Figure 5:**
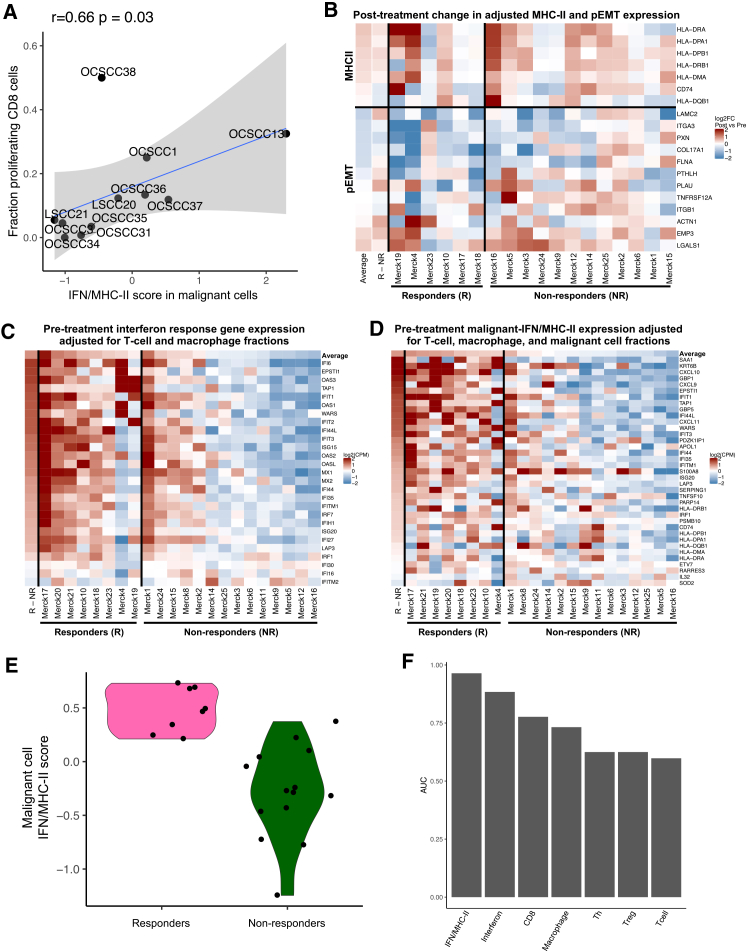
MHC-II upregulation in malignant cells correlates with response to anti-PD1 treatment (A) Scatterplot shows, for each pre-treatment sample, the average expression of the immune metaprogram in malignant cells (*x* axis) and the fraction of proliferating CD8 T cells (*y* axis). Spearman correlation and *p* value are shown. (B) Heatmap shows the difference in gene expression after treatment for 18 bulk samples with paired data. The MHC-II genes are all MHC-II genes included in the malignant-IFN/MHC-II metaprogram, while the p-EMT genes are all genes in the p-EMT metaprogram that were recurrently downregulated in the single-cell data. MHC-II gene expression was adjusted for inferred tissue expression of macrophages and dendritic cells, and p-EMT gene expression was adjusted for expression of fibroblasts and myofibroblasts. (C) Heatmap shows, for all 22 pre-treatment bulk samples, the expression of interferon-response genes, adjusted for inferred fractions of T cells and macrophages. (D) Heatmap shows, for all 22 pre-treatment bulk samples, the expression of genes in the malignant-IFN/MHC-II metaprogram, adjusted for inferred fractions of T cells, macrophages, and malignant cells. (E) Violin plot shows adjusted malignant-IFN/MHC-II program scores for all 22 pre-treatment bulk samples split by response. (F) Bar plot shows AUC values of ROC curves for potential predictors of outcome.

While the malignant-IFN/MHC-II program was consistently upregulated post-treatment regardless of responder status (Figures 2D–2F), the association between malignant cell-specific MHC-II expression and anti-PD-1 response in murine models led us to ask whether malignant cell MHC-II may serve as a biomarker of response in patients. In pre-treatment RNA-seq samples, we discovered that MHC-II genes were upregulated in the malignant cells of responders compared to non-responders (Figure S5A). We also observed that, on the patient level, average program scores for the malignant-IFN/MHC-II signature in pre-treatment malignant cells significantly correlated with the fraction of proliferating CD8^+^ T cells per sample (Pearson’s r = 0.66, *p* < 0.05) (Figure 5A), as well as with expression of MHC-II programs in both endothelial cells and macrophages (Figures S5B and S5C), further supporting the association of MHC-II with a favorable immune microenvironment for PD-1 response.

As these findings were in a limited number of available samples, we sought to validate them in an alternate, orthogonal dataset. While other bulk RNA-seq datasets have shown that pre-treatment IFN signature expression correlates with response to anti-PD-1 therapy,^30^^,^^35^^,^^36^ we specifically sought to assess whether pre-treatment *malignant cell* expression of IFN/MHC-II genes was associated with response. We thus turned to an additional bulk RNA-seq dataset of HNSCC patients treated with neoadjuvant pembrolizumab,^36^ distinct from our scRNA-seq dataset. This cohort consisted of 43 pre- and post-treatment samples taken from 25 patients (8 responders and 17 non-responders).^36^ In this dataset, we attempted to infer the expression of MHC-II genes in malignant and stromal cells by taking the residual expression after controlling for predicted fractions of macrophages and dendritic cells using CIBERSORTx (STAR Methods), along with our own single-cell data to train the deconvolution model.

First, we sought to replicate our findings on treatment-induced gene expression changes in 18 matched pre- and post-treatment samples. Consistent with our previous findings, we found that post-treatment samples, following CIBERSORTx normalization, had modestly but significantly (log2FC = 0.28, *p* < 0.05, one-sample *t* test) higher expression of MHC-II genes compared to pre-treatment samples, regardless of outcome (Figures 5B and S5D). Likewise, while p-EMT genes, normalized by abundance of fibroblasts and myofibroblasts, showed more diverse expression, they were, on average, significantly down-regulated upon treatment (log2FC = −0.2, *p* < 0.05, one-sample *t* test). Interestingly, the downregulation was much stronger in responders than non-responders (log2FC = 0.46, *p* < 0.05, *t* test) (Figures 5B and S5D).

Next, we focused on pre-treatment samples. We noted that the expression of IFN-response genes was significantly increased (log2FC = 1.13, *p* < 0.01, *t* test) in responders (Figure S5E), even when controlling for expected expression by macrophages and T cells (log2FC = 0.94, *p* < 0.01, *t* test) (Figures 5C and S5F). We thus examined the predictive significance of the full malignant-IFN/MHC-II program, which comprises both MHC-II and IFN genes and is uniquely expressed in the malignant cells in our single-cell data, by estimating the specific expression profile of the malignant cells from bulk expression profiles. To this end, we adjusted the bulk expression profiles by subtracting the expected contribution of the macrophages and T cells and normalized the resulting profiles by the purity of the bulk samples (STAR Methods). In these deconvolved data, we noted a significant upregulation of the malignant-IFN/MHC-II program (log2FC = 0.77, *p* < 0.0001, *t* test) in the pre-treatment samples of responders compared to non-responders (Figures 5D and 5E).

Using these adjusted expression values, we compared the malignant-IFN/MHC-II program both to the adjusted general IFN signature (Figure 5C) and to fractions of cell types representing potential sources of IFN. We created receiver operating characteristic (ROC) curves for each of these metrics against responder status and found that pre-treatment expression levels of MHC-II and IFN-response genes by malignant cells stratified patients by immunotherapy response (area under the curve [AUC], 0.96) in this dataset, outperforming the fraction of CD8^+^ T cells (AUC, 0.78) or the overall tissue IFN-response expression (AUC, 0.87) (Figures 5F and S5G). Accordingly, in a combined linear model for prediction of outcome by various features (malignant-IFN/MHC-II signature, overall IFN-response and inferred fractions of macrophages, CD8, T helper cells, and Tregs), only the malignant-IFN/MHC-II signature was significant (*p* < 0.05, *t* test) (Table S4).

## Discussion

We performed scRNA-seq on 137,020 cells from 16 pre- and post-immunotherapy HNSCC patients to investigate cellular changes following PD1 checkpoint blockade. This dataset is among the largest pre- and post-immunotherapy datasets to date and importantly, provides unique insights into the entire tumor ecosystem including malignant cells. Notably, this phase 2 cohort formed the basis for the recent KEYNOTE-689 phase 3 study, which established perioperative pembrolizumab as a new standard of care, offering unique biological insights into this emerging treatment paradigm.

We found that following neoadjuvant pembrolizumab treatment, T cells became enriched with CD8^+^ over CD4^+^ subsets and showed increased representation of specific states. While these results are somewhat consistent with prior studies,^7^^,^^8^ our work suggests that patients exhibited these effects regardless of clinical response. These findings indicate that, at least in HNSCC, anti-PD1 treatment induces a T cell response in most or all patients, but only in a subset of the patients is such a molecular response borne out as an effective clinical benefit.

We then turned to the malignant cells, in which we identified a malignant-IFN/MHC-II program, consisting primarily of MHC-II genes and IFN-response genes, which was enriched following treatment. In a murine model of HNSCC, we demonstrated that while malignant cell MHC-II itself was dispensable for response to anti-PD-1 therapy, it was induced by IFN-γ signaling, and this signaling was required for therapeutic response, potentially through maintenance of a T cell-infiltrated microenvironment. Finally, in pre-treatment samples, the malignant-IFN/MHC-II program was predictive of immunotherapy response. While further study is certainly required in larger prospective cohorts to validate these findings, this signature holds substantial promise for enabling more precise patient stratification prior to immunotherapy.

Overall, these data suggest that the malignant-IFN/MHC-II program may be indicative of not only the presence of T cells within the tumor but also their functional interaction with malignant cells through IFN signaling. Pre-treatment expression of MHC-II genes in malignant cells may reflect an active endogenous anti-tumor immune response, which becomes amplified by anti-PD1 treatment, leading to favorable therapeutic outcomes. Similar findings that (1) pre-treatment expression of IFN signatures (including MHC-II) correlate with response to anti-PD1 therapy^35^ and (2) IFN/MHC-II signatures increase significantly post-treatment in both responders and non-responders have been observed in bulk-RNA-seq studies of other tumor types.^30^^,^^37^ Moreover, based on bulk approaches in triple negative breast cancer, MHC-II expression by malignant cells was associated with increased tumor lymphocyte infiltration and improved prognosis.^38^ In lung adenocarcinoma and melanoma, MHC-II expression by malignant cells was associated with response to anti-PD1 therapy.^39^^,^^40^^,^^41^^,^^42^ Studies in melanoma specifically provide support for the association between MHC-II expression by malignant cells and IFN-γ-mediated signatures^41^ and suggest that this MHC-II expression may be regulated by the Hippo signaling pathway.^42^ However, this prior work was largely limited to bulk approaches or assays in cell lines. Our single-cell analysis of patient samples extends these previous findings to HNSCC and demonstrates that IFN signaling and MHC-II expression arise from malignant cells in patients who are more likely to respond to therapy.

An alternative, less likely, possibility is that MHC-II expression by malignant cells is not merely a proxy for malignant cell-immune cell interactions, but rather that it plays a direct functional role in antigen presentation. While productive antigen presentation by MHC-II is typically done by professional antigen-presenting cells such as dendritic cells and macrophages, accumulating evidence supports the functional significance of MHC-II antigen presentation by epithelial cells in normal physiology,^43^^,^^44^ and possibly also in cancer.^45^^,^^46^ However, our animal experiments suggest that malignant cell-specific MHC-II expression may serve as a marker—but not a functional driver—of immunotherapy response in HNSCC. Of course, acknowledging that alternate mechanisms may be at play in animal models and humans, additional work is needed to directly test the degree to which MHC-II expression by epithelial malignant cells results in effective antigen presentation and the downstream events that may be triggered.

### Limitations of the study

Limitations of the present work include a modest sample size, the use of bulk RNA-seq data for validation, and the use of treatment-naive patients. The modest sample size stems from the limited number of on-treatment patients who were studied while on a phase 2 clinical trial of neoadjuvant immunotherapy, which was further compounded by the paucity of post-treatment tissue remaining for those with a major pathologic response, ultimately limiting stratification of responders in a more nuanced manner. This limitation particularly affected the CODEX analysis, where only a small number of patients had available tissue for analysis. We, therefore, attempted to address sample size limitations using validation in a larger bulk RNA-seq cohort. However, drawing conclusions from the latter is imperfect given the need for deconvolution, although this limitation is mitigated by the consistency of our findings with our scRNA-seq data as well as data from other cancer types. In addition, our mechanistic studies were performed in a single, well-characterized murine HNSCC model (MOC1) grown subcutaneously rather than orthotopically. While this approach enabled highly reproducible tumor growth and longitudinal profiling, it may not fully recapitulate the local tumor-immune interactions of the native oral cavity; thus, future *in vivo* studies in additional models in the oral cavity are needed. Finally, our mechanistic work in a murine model of HNSCC suggests a dominant role for IFN-γ in inducing malignant cell-intrinsic MHC-II expression; however, we cannot rule out a role for TNF or type I IFNs in this process as well. Thus, this work provides a foundation for larger prospective studies aimed at translating these findings into clinical practice.

## Resource availability

### Lead contact

Requests for further information and resources should be directed to and will be fulfilled by the lead contact, Sidharth Puram (sidpuram@wustl.edu).

### Materials availability

This study did not generate new unique reagents.

### Data and code availability

• Processed scRNA-seq data were deposited at GEO: GSE301741; raw data were deposited at SRA: PRJNA1283925. All embargoes have been removed, and datasets are fully public.
• This paper does not report original code.
• Immunofluorescence and mouse data reported in this paper will be shared by the lead contact upon request. Any additional information required to reanalyze the data reported in this paper is available from the lead contact upon request.

## Acknowledgments

Funding: 10.13039/100001368V Foundation (S.V.P.), 10.13039/100002002Cancer Research Foundation (S.V.P.), 10.13039/100007338Barnes-Jewish Hospital Foundation (S.V.P.), 10.13039/100000862Doris Duke Foundation (S.V.P.), 10.13039/100000072NIDCR 1R01DE032371 (S.V.P.), NIDCR 1R01DE032865 (S.V.P.), NIDCR 1K08DE033093 (A.S.P.), 10.13039/501100003977Israel Science Foundation (to I.T.), and 10.13039/100000054NCI 5T32CA009547-38 (J.M.Z.). I.T. is the incumbent of the Dr. Celia Zwillenberg-Fridman and Dr. Lutz Zwillenberg Career Development Chair and is supported by the Zuckerman STEM Leadership Program. We thank generous philanthropic support from the Siteman Head and Neck Cancer Fund and The Robert Ebert and Greg Stubblefield Head and Neck Tumor Center. The work was also supported by the Bursky Center for Human Immunology and Immunotherapy Programs at 10.13039/100007268Washington University, Immunomonitoring Laboratory. The funding sources had no involvement in the design, conduct, or reporting of the research. Figure 1A and the graphical abstract were created in BioRender (Parikh A., 2025, https://BioRender.com/wc0k1q4).

## Author contributions

M.M., R.S., A.S.P., J.M.Z., D.R.A., R.U., I.T., and S.V.P. designed and executed the study and wrote and edited the manuscript. Z.Q., T.L., F.W., T.F.B., R.M., E.S., A.R., S.R., S.G., P.O., and J.L. performed data analysis and edited the manuscript. W.T., R.C.P., J.T.R., R.A.H., P.A.Z., R.S.J., and P.P. provided patient samples and edited the manuscript.

## Declaration of interests

R.U. reports grants and personal fees from Merck, Regeneron, and Daichi-Sankyo. The MOC models developed by R.U. have been filed with the Washington University Office of Technology Management and are licensed for distribution by Kerafast.

## STAR★Methods

### Key resources table

**Table:** 

REAGENT or RESOURCE	SOURCE	IDENTIFIER
**Antibodies**		

CCXCR1/XCR1, clone CIA207A	Abcam	Cat# ab317579, RRID AB_3740932
CD20, clone L26	Akoya	Cat# 4450018; RRID AB_2915939
PDL1, clone 73-10	Akoya	Cat# 4550128; RRID AB_3676534
FOXP3, clone AKYP0102	Akoya	Cat# 4550071; RRID AB_2927679
Ki67, clone B56	Akoya	Cat# 4250019; RRID AB_2895046
LAMC2, clone EPR23654-127	Abcam	Cat# ab274384, RRID AB_3740933
Pan-Cytokeratin, clone AE-1/AE-3	Akoya	Cat# 4450020; RRID AB_3083456
TOX1, clone AKYP0098	Akoya	Cat# 4250067; RRID AB_3477620
CD31, clone EP3095	Akoya	Cat# 4450017; RRID AB_2915935
NKG2A, clone EPR23737-127	Abcam	Cat# ab273516; RRID AB_2943182
CD163, clone EPR19518	Akoya	Cat# 4250079; RRID AB_2935895
CD8, clone C8/144B	Akoya	Cat# 4250012; RRID AB_2915960
PD-1, clone AKYP0070	Akoya	Cat# 4550038; RRID AB_3096407
CD4, clone EPR6855	Akoya	Cat# 4350018; RRID AB_2915936
SMA, clone 1A4	Akoya	Cat# 4450049; RRID AB_2936084
Granzyme B, clone AKYP0088	Akoya	Cat# 4250055; RRID AB_3472025
CD45RO, clone UHCL1	Akoya	Cat# 4250023; RRID AB_2895053
CD11c, clone 118/A5	Akoya	Cat# 4550114; RRID AB_3083459
CD14, clone AKYP0079	Akoya	Cat# 4450047; RRID AB_3083457
IFNgamma, clone AKYP0093	Akoya	Cat# 4250062; RRID AB_3476455
HLA-DR, clone EPR3692	Akoya	Cat# 4450029; RRID AB_2928988
CD3e, clone EP449E	Akoya	Cat# 4550119; RRID AB_2936080
TCF-1, clone AKYP0099	Akoya	Cat# 4250067; RRID AB_3477620
CD68, clone KP1	Akoya	Cat# 4350019; RRID AB_2935894
IDO1, clone VINC3IDO	Akoya	Cat# 4550123; RRID AB_3476035
CD16a, clone SP175	Abcam	Cat# ab243925; RRID AB_3697253
TP63, clone AKYP0111	Akoya	Cat# 4550081; RRID AB_3713478
HLA-A, clone AKYP0078	Akoya	Cat# 4450046; RRID AB_3678453
Granzyme K, clone EPR24601-164	Abcam	Cat# ab282714; RRID AB_3665531
Anti-PD1 monoclonal antibody	BioXcell	Cat# BE0146; RRID AB_10949053
Anti-trinitrophenol isotype-matched control	BioXcell	Cat# BE0089; RRID AB_1107769
Anti-IFN-γ monoclonal antibody, clone H22	Leinco	Cat# I-438; RRID AB_2737542
Armenian Hamster IgG Isotype Control, clone PIP	Leinco	Cat# I-140; RRID AB_2737537
BV 650 anti-mouse PD-L1, clone B7-H1	Biolegend	Cat# 124336; RRID AB_2734192
APC-Cyanine 7 anti-mouse MHC Class II (I-A/I-E), clone M5/114.15	Cytek	Cat# 25-5321-U025, RRID AB_3740934
PE anti-STAT1 phospho (Ser727), clone A15158B	Biolegend	Cat# 686403; RRID AB_2616938
violetFluor 500 anti-mouse CD45, clone 30-F11	Cytek	Cat# 85-0451-U025; RRID AB_3695656
cFluor V610 anti-mouse CD4, clone RM4-5	Cytek	Cat# R7-20270; RRID AB_3674605
FITC anti-mouse CD8, clone 53-6.7	Cytek	Cat# 35-0081-U025; RRID AB_2621671
PE-Cyanine7 anti-mouse CD11b, clone M1/70	Cytek	Cat# 60-0112-U025; RRID AB_2621836
violetFluor 450 anti-mouse Ly-6G, clone 1A8	Cytek	Cat# 75-1276-U025; RRID AB_2621955
PerCP-Cyanine5.5 anti-mouse Ly-6C, clone HK1.4	Cytek	Cat# 65-5932-U025; RRID AB_3674607
PE anti-mouse F4/80, clone BM8	Cytek	Cat# 50-4801-U025; RRID AB_2621795
PE anti-mouse H-2, clone M1/42	Biolegend	Cat# 125505; RRID AB_1227706
BV 786 anti-mouse EpCAM, clone G8.8	ThermoFisher	Cat# 417-5791-80; RRID AB_3074147

**Biological samples**		

Human head and neck squamous cell carcinoma patient samples	Washington University	N/A

**Chemicals, peptides, and recombinant proteins**		

Mouse IFN-gamma Recombinant Protein	PeproTech	Cat# 315-05

**Critical commercial assays**		

gentleMACS Octo Dissociator	Miltenyi	Cat# 130-096-427
Chromium Single Cell 3’ (V2 Chemistry) platform	10X Genomics	Cat# PN-120237
Chromium Single Cell 5’ (V2) platform	10X Genomics	Cat# PN-1000263
MERCURIUS BRB-seq kit	Alithea Genomics	Cat# 10813

**Deposited data**		

Processed single cell RNA-seq data	This paper	GEO: GSE301741
Raw single cell RNA-seq data	This paper	SRA: PRJNA1283925
Raw bulk RNA-seq data	Uppaluri et al.^36^	dbGaP: phs002864

**Experimental models: Cell lines**		

Mouse oral cavity 1 (MOC1) cell line	Kerafast	EWL001-FP

**Experimental models: Organisms/strains**		

C57BL/6 mice	Jackson Laboratories	000664

**Oligonucleotides**		

Ifngr1 Alt-R CRISPR-Cas9 sgRNA: GTACCGACGAATGTTCTAAT	IDT	N/A
Ciita Alt-R CRISPR-Cas9 sgRNA: GAGCGCCAGCTAGCCCACGGNGG	IDT	N/A

**Software and algorithms**		

CellRanger v3.1.0	10X Genomics	https://github.com/10XGenomics/cellranger
CellBender v0.2	Fleming et al.^47^	https://cellbender.readthedocs.io/en/latest/introduction/index.html
scAtlasVAE	Xue et al.^19^	https://scatlasvae.readthedocs.io/en/latest/
CIBERSORTx	Steen et al.^48^	https://cibersortx.stanford.edu/
QuPath v0.6.0-rc3	Bankhead et al.^49^	https://qupath.readthedocs.io/en/0.6/
InstanSeg	Goldsborough et al.^50^	https://github.com/instanseg/instanseg
FlowJo v10.10.0	FlowJo, LLC	https://www.flowjo.com/flowjo/overview
R v4.3.1	R Core Team^51^	https://www.R-project.org/
NMF	Gaujoux et al.^52^	https://github.com/renozao/NMF
STARsolo v2.7.6a	Kaminow et al.^53^	https://github.com/alexdobin/STAR/blob/master/docs/STARsolo.md
Jupyterlab v4.0.11	Kluyver et al.^54^	https://github.com/jupyterlab/jupyterlab
Seaborn v0.13.2	Waskom et al.^55^	https://pypi.org/project/seaborn/
Scanpy v1.10.1	Wolf et al.^56^	https://pypi.org/project/scanpy/
scimap v0.14	Nirmal et al.^57^	https://scimap-doc.readthedocs.io/en/latest/

### Experimental models and subject details

#### Human subjects and study design

This study investigated samples collected from patients in a phase 2 clinical trial (NCT02296684) of neoadjuvant pembrolizumab in patients with locally advanced Stage III/IVB surgically resectable head and neck squamous cell carcinoma (HNSCC). The study was conducted in accordance with the Declaration of Helsinki, was approved by the Institutional Review Board (IRB) of Washington University (#201412118). Briefly, patients with resectable clinical stage III to IVB HNSCC without distant metastases were treated with two doses (cohort 2) of pembrolizumab (200mg, intravenous, every 3 weeks) prior to curative-intent surgical resection and standard-of-care adjuvant therapy. Pathologic response was defined as area of tumor regression and assessed independently by two head and neck pathologists before consensus scoring. Responders was classified as pTR-2 (>50%) or pTR-1 (10–50%) tumor regression, while <10% was considered non-response. The full protocol, trial design, patient characteristics, and clinical outcomes were recently reported^7^ and include the 16 patients in this study. Tumor specimens for scRNA-seq were collected at baseline before neoadjuvant therapy, and at the time of surgical resection at Washington University (under IRB #201102323) and have not been previously reported.

#### MOC1 cell line and cell culture

The MOC1 oral cavity squamous cell carcinoma line was kindly provided by Dr. Ravindra Uppaluri (Dana-Farber Cancer Institute) and authenticated by genotyping of strain-specific alleles. The MOC1 cell line was derived from a squamous cell carcinoma tumor from a C57BL/6 mouse administered 25 weeks of oral DMBA exposure.^26^ MOC1 were cultured in IMDM-based MOC medium consisting of IMDM (Gibco, 12440061), Ham’s Nutrient Mixture F10/F12 (Gibco, 11765047), 5% fetal bovine serum (Peak Serum, PS-FB1), 100 U/mL penicillin–streptomycin (Gibco, 15140122), 5 ng/mL EGF (Thermo Fisher, 01107MI), 400 ng/mL hydrocortisone (Sigma, H0888), and 5 μg/mL insulin (Sigma, I0516) at 37°C. Cell lines were tested monthly for mycoplasma contamination.

#### Murine model of HNSCC

Female C57BL/6J mice (#000664) were purchased from The Jackson Laboratory and housed in a specific pathogen-free barrier facility maintained by the Washington University School of Medicine Division of Comparative Medicine. Mice were 6–7 weeks of age at the time of tumor implantation. All animal experiments were conducted in accordance with protocols approved by the Institutional Animal Care and Use Committee (IACUC) at Washington University School of Medicine. Only female mice were used to avoid housing issues and aggression associated with male mice; therefore, sex was not evaluated as a biological variable in this study.

MOC1 cells were resuspended in phosphate-buffered saline and injected into the flanks of WT C57BL/6 mice at a final concentration of 5 × 10^6^ cells/0.15 mL per animal. Tumor sizes were measured every 2 days with digital calipers. Mice were monitored for signs of morbidity and tumor ulceration, and animals reaching endpoints set by the Washington University Institutional Animal Care and Use Committee (IACUC) protocol associated with this study were humanely euthanized. All animals were euthanized on day 30 post-tumor engraftment. For immunotherapy, MOC1 tumor-bearing mice received 250 μg per animal per treatment of anti-PD1 monoclonal antibody (BioXcell, BE0146) or anti-trinitrophenol isotype-matched control monoclonal antibody (BioXcell, BE0089) on days 3, 6, and 9 post-engraftment via intra-peritoneal (IP) injection. For IFN-γ neutralization, mice were administered 200 μg of hamster anti-IFN-γ monoclonal antibody (clone H22, gift from the laboratory of Robert Schreiber, Washington University School of Medicine) or isotype-matched control antibody (clone PIP, gift from the laboratory of Robert Schreiber, Washington University School of Medicine) every 7 days via IP injection starting 7 days prior to tumor engraftment.

#### *Ifngr1* and MHC-II-deficient MOC1 cell lines

MOC1 cells underwent CRISPR/Cas9-mediated knockout (KO) of *Ifngr1* or *Ciita* by nucleofection of SpCas9 protein (IDT, 1081058) complexed with single-guide RNA (sgRNA) targeting exonic regions present in all isoforms of murine *Ifngr1* or *Ciita*. Briefly, 1 × 10^4^ MOC1 cells were nucleofected with Cas9-sgRNA complexes using the Lonza 4D-Nucleofector X Unit (EN158 protocol) and the P3 Primary Cell 4D-Nucleofector X Kit S (Lonza, V4XP-3032). Optimal nucleofection conditions were determined using the pmaxGFP Control Vector (Lonza). Successful nucleofection was confirmed by assessing GFP expression via fluorescence microscopy 24 h post-nucleofection. Control cell lines were generated by nucleofecting Cas9 protein alone. Editing at the targeted locus was also confirmed via targeted next-generation sequencing. The following targeting sequences were used to create sgRNAs in the Alt-R CRISPR-Cas9 system (IDT):

sgIfngr1: GTACCGACGAATGTTCTAAT

sgCiita: GAGCGCCAGCTAGCCCACGGNGG.

### Method details

#### Sample processing and sequencing

Fresh biopsies were collected from primary tumor sites and placed in ice-cold Dulbeco’s Modified Eagle Medium (DMEM, Thermo Fisher). Samples were minced into 2-3mm fragments, washed in PBS (Thermo Fisher) and dissociated using a Human Tumor Dissociation Kit (Miltenyi Biotec #130-095-929) per manufacturer guidelines. Samples were dissociated at 37°C for 1 h on a gentleMACS Octo Dissociator (Miltenyi, #130-096-427) or agitated manually by pipette every 10 min if initially smaller than 4 × 4mm. Cell suspensions were passed through a 40 μm filter (Thermo Fisher, #22-363-547), centrifuged at 450g for 5min (as was all cell pelleting unless otherwise noted), followed by Ammonium-Chloride-Potassium red blood cell lysis if pellet remained bloody (per manufacturer protocol, Thermo Fisher, #A1049201). Dissociated cells were spun, then resuspended in AutoMACS Rinsing Solution with 0.5% BSA (Miltenyi Biotech). The single-cell suspension was sorted via magnetic column using either human CD45 magnetic MicroBeads (Miltenyi) or human CD3 magnetic MicroBeads (Miltenyi) according to manufacturer’s protocol for up to 10^7^ total cells. The positive and negative cell fractions were then spun, counted, and mixed in a 1:2 ratio of positive:negative fractions to enrich for malignant/stromal cells. For one patient OCSCC1 (pre and post), both CD45^+^ and CD45^−^cell fractions were sequenced separately. Samples with less than 10^6^ cells were not sorted. Samples were processed using either the Chromium Single Cell 3’ (V2 Chemistry) or 5' (V2) platform with a target of ∼10,000 cells (10x Genomics) following the manufacturer’s instructions. Briefly, cells were added onto a chip to form Gel Bead-in-Emulsions, followed by cell lysis, reverse transcription, tagmentation, adapter ligation and addition of sample index to the libraries before sequencing. scRNA-seq libraries were sequenced on Illumina NovaSeq machines with a target minimum read count of 0.5 billion per sample.

#### CODEX immunofluorescence staining

Freshly sectioned human FFPE tissue slides were baked for 90 min at 65°C in a HybEZ II Oven (Advanced Cell Diagnostics, USA) immediately prior to dewaxing. Deparaffinization and rehydration in 100%, 70% and distilled water were performed on the Parhelia Spatial Station automated platform using their proprietary dewaxing agent. Antigen retrieval was performed in 1X Tris-EDTA pH 9.0 (Invitrogen 00-4956-58, Thermo Fisher Scientific, USA) in the chamber at 108°C for 30 min per Parhelia Spatial Station protocol. The staining chamber was allowed to cool to 30°C before rinsing the slides in distilled water twice, then transferring Akoya Hydration Buffer for 2 min before washing again in Hydration buffer. The Akoya Staining Buffer was applied for 20 min prior to the antibody mix addition in the Parhelia Spatial Station. Slides were incubated in 120 μL of antibody mix at room temperature in a humidified chamber for 3 h. After incubation, the slides were washed and fixed according to the Akoya PhenoCycler-Fusion User Guide_1.0.3_ RevD protocol. The slides were then manually photobleached for 45 min in 20mM NaOH/4.5% Hydrogen peroxide/PBS solution while sandwiched between two LED lights as described by Du, Z et al. Nat Protoc (2019). After photobleaching, the flow cells were applied and the slides were imaged on an Akoya PhenoCycler Fusion 2.0 co-detection by indexing (CODEX) multiplex imaging system (Akoya Biosciences, USA). Images were collected as 8-bit QPTIFF files with embedded metadata. Non-Akoya antibodies were ordered carrier-free from their respective manufacturers and conjugated to the indicated Phenocycler DNA barcodes according to manufacturer instructions. All above deparaffinization, staining, and imaging were performed in the WashU Immunomonitoring Laboratory (IML) Core.

#### Cell sorting for CRISPR knockout selection

4 days post-nucleofection, control and KO cell line pools were treated with 100 ng/mL IFN-γ (PeproTech, 315-05) to induce expression of PD-L1 (for *Ifngr1* KO) or MHC-II (for *Ciita* KO). Following 6 h (*Ifngr1* KO) or 48 h (*Ciita* KO) of IFN-γ treatment, control and KO pool cell lines were stained for phospho-STAT1 (BioLegend, BV 650 anti-mouse PD-L1 clone 10F.9G2, 124336) or MHC-II (Cytek, APC-Cyanine7 Anti-Mouse MHC Class II (I-A/I-E) clone M5/114.15.2, 25–5321). Stained cells then underwent negative selection via FACS sorting on a CytoFLEX SRT Benchtop Cell Sorter (Beckman Coulter). KO was confirmed by sampling each cell line and re-exposing to IFN-γ for 6 h (*Ifngr1* KO) or 48 h (*Ciita* KO), followed by flow cytometry analysis for phospho-STAT1 (BioLegend, PE anti-STAT1 Phospho (Ser727) Antibody clone A15158B, 686403) or MHC-II expression. Given the minor role of *Ciita* in regulating MHC-I expression,^33^ *Ciita* KO cell lines were also assessed for MHC-I expression (BioLegend, PE anti-mouse H-2 Antibody, clone M1/42, 125505) to confirm no significant effect on class I expression. After confirmation of gene editing, WT or KO cell lines were injected into animals according to the above protocol.

#### Mouse tumor sample processing

MOC1 tumors were dissected, minced, washed with PBS (Thermo Fisher), and dissociated using the Mouse Tumor Dissociation Kit (Miltenyi Biotec, 130-096-730) per manufacturer protocol. Dissociated cell suspensions were passed through a 70 μm strainer (MIDSCI, 70ICS) and washed with PBS supplemented with 0.5% BSA (Miltenyi Biotec, 130-091-376). Washed cells underwent red blood cell lysis (Thermo Fisher, 00-4300-54) for 3 min on ice. Lysis was quenched with 12 mL of PBS +0.5% BSA and cells were pelleted and resuspended in 1 mL of PBS plus 0.5% BSA and kept on ice for further processing and analysis.

#### Flow cytometry analysis

For MOC1 tumors, live cells were distinguished by staining with Zombie Yellow Fixable Viability Dye (BioLegend, 423103) in PBS at 1:4000 dilution for 20 min at 4°C. Cell suspensions were washed and pelleted. Surface staining for tumor and immune cells was then performed for 30 min at 4°C in PBS +0.5% BSA. Cell suspensions were washed twice with PBS and resuspended in PBS +0.5% BSA. Flow cytometry data was acquired immediately after sample processing.

For *in vitro* MOC1 lines, adherent cells were detached after a brief incubation in 0.25% trypsin-EDTA (Gibco, 25200114). Cell suspensions were rinsed once with serum-containing media and twice with ice-cold PBS. Cell pellets were re-suspended in 1 mL of PBS +0.5% BSA and underwent staining according to the above protocol.

For intracellular staining of phospho-STAT1, live cells were fixed at 37°C for 15 min in pre-warmed Fixation Buffer (BioLegend, 420801). Fixed cells were washed 3 times in PBS +0.5% BSA and subsequently permeabilized in pre-chilled True-Phos Perm Buffer (BioLegend, 425401) at −20°C for 1 h. Fixed and permeabilized cells were washed three times with PBS +0.5% BSA and underwent staining.

Fluorescent-labeled antibodies against mouse CD45 (30-F11), I-A/I-E (M5/114.15.2), CD3 (145-2C11), CD4 (SK3), CD8 (SK1), CD11b (M1/70), Ly-6G (1A8), and Ly-6C (HK1.4) were obtained from Cytek as part of the Tonbo Myeloid Identification Kit. Antibodies against mouse F4/80 (BM8) and H-2 (M1/42) were obtained from BioLegend. Antibody against mouse PD-L1 (B7-H1) was obtained from Cytek. Antibody against mouse EpCAM (G8.8) was obtained from Thermo Fisher.

Flow cytometry data were acquired using the Cytek Northern Lights (Cytek) full-spectrum profiling system. Optimal antibody combinations were determined using Cytek Cloud Panel Builder (Cytek) to minimize fluorophore spectral overlap. For all experiments, fresh unstained and single-stained controls were generated by pooling cells from each sample. Spectral unmixing was performed per manufacturer recommendations. Flow cytometry data were analyzed using FlowJo version 10 software. For experiments reporting fold changes in mean fluorescence intensity (MFI), values were normalized to the mean MFI of the reference condition, such that the reference mean MFI equals 1.0.

#### BRB-seq analysis of mouse tumors

Bulk RNA barcoding and sequencing (BRB-seq) is a highly scaled RNA-sequencing method that employs a sample barcoding step during reverse transcription. This early barcoding step allows for the pooling of all samples prior to library construction. This streamlined approach reduces library preparation costs and labor, which permits the multiplexing of up to 96 libraries per experiment.^58^ Total RNA from MOC1-derived tumors was obtained from processed cell suspensions using the RNeasy Mini Kit (Qiagen, 74104) with an additional on-column 15 min DNase I (Qiagen, 79254) digestion. RNA concentrations were determined using an Implen N50 NanoPhotometer (Cole-Parmer). Library preparation was performed using the MERCURIUS BRB-seq kit (Alithea Genomics). Library sequencing was performed to a depth of 12 million reads per sample on the Illumina NovaSeq X Plus with the following setup: Read 1 = 150 cycles, Index 1 = 10 cycles, Read 2 = 150 cycles, Index 2 = 10 cycles. Adapter trimming was performed using Cutadapt (v2.10).

### Quantification and statistical analysis

#### scRNA-seq alignment and doublet detection

After sequencing, FASTQ files were aligned to a pre-built human reference genome hg19 (10X Genomics, July 24, 2019) using CellRanger v3.1.0 (10X Genomics) ‘count' command with default options. Ambient RNA was removed using CellBender v0.2,^47^ with manually tuned hyper-parameters z-dim, z-layers, learning-rate, and expected-cells to maximize training stability. For each sample, cells with fewer than 500 detected genes were removed as low-quality, and genes detected in less than 0.1% of observed cells were discarded. Doublets were identified using Scrublet v0.2.3, with the following modifications. Hyperparameters for expected_doublet_rate and n_neighbors were automatically determined using an automated grid search seeking to maximize the separation of simulated self-self doublets and self-other doublets. Thresholding was determined based targeting a false negative rate of 1%, as determined by the simulated self-self doublets. Finally, to rescue rare samples which had been overloaded, 30% of cells considered by Scrublet were randomly sampled from all other samples, omitting the overloaded samples. Following doublet removal, samples were over-clustered using the scanpy leiden function with a resolution of 1, and cells from each cluster above 2 standard deviations of the mean % mitochondrial reads and % ribosomal reads were removed from further consideration.

#### Quality control and preprocessing

Transcriptomic data underwent normalization by converting UMI counts to CPM (counts per million), calculated by dividing each gene’s count by the sample’s total sum of UMIs. This was followed by transformation using the formula log_2_(CPM/10 + 1). Centering was applied by subtracting each gene’s mean expression value from all observations for that gene. Only genes that either maintained a mean expression of ≥ 4 log_2_(CPM) across the entire dataset or had ≥ 5 UMI counts in a minimum of 20 cells were retained for downstream analyses.

Each cell was initially assigned a cell type by scoring for canonical markers. All cells with a non-lymphoid initial cell type that expressed fewer than 1000 genes were removed as low-quality, as were lymphoid (B-cells, T-cells, NK-cells and plasma cells) cells with fewer than 500 expressed genes due to these cells’ lower complexity. Additionally, cells with more than 20% of mitochondrial-gene derived UMIs were filtered out as indicative of low quality.

#### Cell type assignment and batch correction

All subsequent processing and analysis of human scRNA-seq data was performed in R (v 4.3.1).^51^ The gene-cell matrix underwent dimension reduction using UMAP and Louvain clustering (k = 200), and all clusters were assigned to a cell type based on their top 50 differentially expressed genes compared to other clusters. Batch correction was then applied to correct for both 3′ and 5′ sequencing being used on the same samples. For every cell type cluster and every patient with both 3′ and 5′-sequenced samples, the expression values of 3′-sequenced cells were centered to the expression values of all 5′-sequenced cells from the same patient and cell type. Final assignments were achieved through dimension reduction and clustering of the corrected matrix.

Cells fulfilling either of the three following conditions: 1) TCR-positive cell in non-T/NK-cell cluster, 2) highest cell signature score discordant with cluster assignment or 3) highest cell signature score less than 1.15∗second highest signature score AND second highest signature scoring cell type discordant with cluster assignment, were set as unresolved and removed from further analysis; 16,245 cells were filtered out using this approach. Assignment to any non-stromal subtype in a stromal cluster was defined as discordant, and likewise for epithelial clusters. Cells classified as fibroblasts in epithelial clusters were retained, as these could indicate malignant cells undergoing EMT. For immune clusters, only cells individually classified to the cluster cell type were considered non-discordant, except T- and NK-cells, which clustered together.

#### Scoring cells for gene signatures

We created gene expression signature scores per cell using our previously described approach.^59^ Relative expression scores for individual cells were created by subtracting the mean expression of the gene signature in a cell by that of a reference gene set. To generate the reference set, we first stratified all genes under analysis into 30 bins based on their mean expression levels, then randomly selected 100 genes from the corresponding expression bin for each signature gene.

#### Differential gene expression analysis

For each differential gene expression analysis, an unfiltered UMI matrix was created from only the relevant cells. When applicable, 3’/5′ batch correction was applied on a per-patient basis as described above. The UMI matrix was then normalized, filtered, transformed and centered as described above. Two-sided t-tests were performed for each gene between the groups in the analysis. *p*-values were adjusted for multiple testing through Benjamini-Hochberg correction.

#### Non-negative matrix factorization

Non-negative matrix factorization (NMF) was used to explore within-cell type heterogeneity. For each cell type studied, samples with at least 30 cells of that type were selected. A new matrix was created from the cells belonging to the cell type studied in each sample. The matrix underwent filtering, batch correction and centering as described above. Negative values were set to zero and NMF was performed using the snmf/r factorization algorithm from the NMF R package.^52^ For each sample and cell type, the algorithm was run 20 times to select the factorization solution with the lowest approximation error.

Matrices were split into ten factors, every factor defined by the top 100 genes by NMF weight. All cells were assigned to the factor whose genes were most highly expressed in that cell. Factors with <10 cells assigned were removed. Jaccard similarities were then calculated between every pair of gene lists from the remaining factors. Factors without a Jaccard overlap ≥ 0.2 with any other factor were removed, representing sample-specific rather than recurrent programs. In malignant cells, a total of 51 factors were thus retained. The Jaccard similarity matrix from the remaining factors underwent hierarchical clustering using Euclidean (1-Jaccard similarity) distance as distance metric, with average linkage.

Metaprograms were created from groups of factors clustering together and representing recurrent biological programs(metaclusters). In every metacluster containing factors from ≥2 patients, genes found in the factors of >50% of patients represented in the cluster were retained as a metaprogram signature and used to assign cell subtype. Cells were assigned to subtypes by creating matrices from all cells of one type across all patients and scoring the cells for metaprogram signatures as described above.

#### Assignment of NK cells

Initially, T- and NK-cells, since they clustered together, were treated as one cell type. NMF and subtype assignment by NMF was applied to all T- and NK-cells together, and all 2,489 cells assigned to the NK-cell metaprogram were considered potential NK-cells or NKT-cells. For each patient with at least 10 5′-sequenced cells in this category, we calculated differential gene expression between cells with and without a TCR and selected the top 50 most overexpressed genes. We then used genes present in at least five comparisons to create signatures for NK and T-cells, respectively (Table S5) and scored all cells for these signatures, assigning them as either T or NK cells.

#### Assignment of T-cells

After the above assignment of NK cells, we were left with 40,746 T-cells. To separate T-cells into CD8, T-helper and T-regulatory subsets, we studied each cell’s expression of the canonical marker genes CD8A, CD8B, CD4, FOXP3 and IL2RA. For a first, stringent, definition, cells with at least one UMI for either CD8A or CD8B, and zero UMIs for the other markers were classified as CD8^+^ T-cells. Cells with at least one CD4 UMI and zero UMIs for all other markers were classified as CD4^+^ T-helper cells, and cells with at least one FOXP3 UMI and one IL2RA UMI, as well as zero CD8A/CD8B UMIs were classified as T-regulatory cells. This initial classification assigned 21,103, or slightly over 50% of T-cells into a subtype. Only these stringently classified cells were used for creating CD8/Th/Treg metaprograms through NMF.

We further clustered all T-cells, removed cells with markers for multiple subsets and reassigned “unresolved” cells, i.e., cells that could not be confidently assigned to any subtype due to lack of marker expression based on a kNN approach, with k set to 10. If at least 8/10 of the stringently assigned nearest neighbors of an unresolved cell belonged to one of the subsets, that cell was reassigned as belonging to the same subset. After this step, 30,377 (75%) of T-cells were assigned to a T cell subset.

#### scAtlasVAE projection

To compare label annotations between our cell subtypes (maximal NMF program per cell) and the cells in the human Antigen Receptor database (huARdb), we used scAtlasVAE.^19^ Reference data was obtained from https://zenodo.org/records/12542577/files/huARdb_v2_GEX.CD8.hvg4k.h5ad, along with the the pre-computed cell latent representation model https://zenodo.org/records/13382785/files/huARdb_v2_GEX.CD8.hvg4k.X_gex.npy. The scatlasvae.pipeline.run_transfer command was used with the reference data, model, our data from CD8 T-cells with an assigned subtype, and latent_key = “cell_type_3” with default parameters.

#### CNA inference

Malignant cells were identified by inferring CNAs from the gene expression data following our previously described approach.^17^ For each patient, a matrix was constructed using all epithelial and stromal cells. The matrix was filtered, normalized, and centered as described above. Genes were ordered by chromosomal position, and normalized expression values were truncated to the range [−3,3]. Copy number profiles were inferred independently for each chromosome by computing a moving average across a sliding window of 100 genes for each position along the chromosome. Mean CNA profiles were calculated for every stromal cell type, generating multiple reference profiles representing diploid cells. For each epithelial cell, CNA values were adjusted by subtracting the highest stromal reference value from positive CNA values and the lowest (negative) reference value from negative values. To further denoise the matrix, CNA values in the range [−0.15,0.15] were set to zero.

#### Subclone assignments

Genetic subclones were inferred from the epithelial cells in the CNA matrix using our previously published approach.^14^ The epithelial CNA matrix was initially filtered to retain the top 2/3 genes by absolute CNA value. We then performed UMAP dimension reduction and overclustering of the UMAP coordinate matrix through Louvain clustering (k = 15). Clusters with <10 cells were merged with their most similar cluster by KNN distance, with k = ln(number of cells). Mean CNA values were then calculated across all cells per cluster and chromosome arm. Copy number events were defined per cluster if the mean CNA signal in the cluster and chromosome arm was < −0.15 or >0.15. Clusters were merged if they: (a) had the same events across all chromosome arms, and (b) the maximum difference between clusters across all chromosome armswas <0.15. Merging continued iteratively, with new mean CNA values calculated and copy number events called, until all remaining clusters differed by at least one chromosome arm.

#### Malignant cell definitions

Malignant and non-malignant epithelial cells were distinguished using two quantitative measures: CNA signal and CNA correlation, computed for each epithelial cell. CNA signal was defined as the mean absolute CNA value across the top two-thirds of genes ranked by absolute CNA value. CNA correlation was calculated as the correlation between the CNA profile of each single cell and the mean CNA profile of the 25% epithelial cells with the highest CNA signal. To avoid differing genetic profiles across the malignant cells in one tumor from impacting malignant/non-malignant assignments, malignant cells were defined separately within each inferred genetic subclone. For each subclone, thresholds for CNA signal and CNA correlation were selected such that fewer than 1% of all stromal reference cells in the patient exceeded each cutoff. Cells exceeding both thresholds were designated malignant, cells failing both were classified as non-malignant epithelial, and cells exceeding only one threshold were labeled as unresolved.

#### CIBERSORTx

CIBERSORTx^48^ was used to infer fractions of cell types in bulk RNA-seq data. As reference matrix, we used a single-cell UMI matrix created from our own data. To improve performance, we decreased the number of cell types through removing cell types with fewer than 1,000 cells and merging pDCs and cDCs into a DC category. For every cell type and sequenced sample with at least 10 cells belonging to that cell type, we randomly sampled maximum 50 cells (lower if sample had 10-49 cells). Only nonzero genes that were also expressed in at least 5 samples from the bulk matrix were kept in this reference matrix. The final reference matrix contained 16,787 cells and 18,769 genes. A CIBERSORTx signature matrix was created through applying the “Create Signature Matrix” command with standard parameters. A bulk mixture matrix was created by using the raw count matrix and only keeping genes present in the signature matrix as well as at least 5 bulk samples. To impute cell fractions, the “Impute Cell Fractions” command was run, disabling quantile normalization and applying S-mode batch correction and 100 permutations.

#### Bulk analysis

To validate and extend our findings from scRNA-seq, we used a bulk RNA-seq dataset of HNSCC patients treated with neoadjuvant pembrolizumab as part of the same phase 2 clinical trial as patients in our scRNA-seq dataset. This cohort consisted of 43 pre- and post-treatment samples taken from 25 patients (8 responders and 17 non-responders).^36^ For analysis of bulk RNA-seq data, the count matrix was log_2_(CPM/10 + 1)-transformed, as described above, keeping only genes with an average expression of >4 log_2_(CPM) across all samples. At this step, no centering was applied. To remove the effect of sample composition on gene expression, we created linear models where we regressed all gene expression scores of interest, against the imputed fractions of confounding cell types (i.e., macrophages and total T cell fractions for the interferon score). Model residuals were used as final scores for downstream analysis. AUC values for outcome analysis were calculated using the pROC R package.^60^

#### CODEX analysis

##### Segmentation of CODEX images

For each slide image, tissue boundaries were identified by pixel classifier using QuPath v0.6.0-rc3^49^ (16.23 μm/px resolution, smoothing 2, threshold 5) on the DAPI channel. Staining or tissue artifacts (i.e., tissue folds, bubbles, reporter aggregates, cautery edge artifacts) were annotated manually and excluded from segmentation and further analysis. Nuclear and cell segmentation were performed with the Instnseg^50^ plugin within QuPath, applying the associated pre-trained model (fluorescence_nuclei_and_cells, CPDMI_2023 dataset^61^). The DAPI, PanCK, TP63, CD3e, CD4, CD8, CD45RO, CD14, CD20, and HLA-A channels were used for cell segmentation. For each cell, mean pixel fluorescent intensity per marker, a unique cell ID, and centroid x and y pixel coordinates were exported in a matrix for further analysis in python (v3.12.3) with Scanpy (v1.10.4).^56^

##### Cell phenotyping of CODEX data

For each image, fluorescent intensity was normalized for each marker by taking the centered log ratio (CLR) of each cell’s mean marker intensity (8-bit, range 0–256) over the geometric mean of all cells in the image, as implemented with pseudocounts in the scverse muon (v0.1) package. Sometimes applied for protein-count data in CITE-Seq,^62^ CLR is similar to the log transformation and *Z* score normalization per sample suggested by Hickey et al.^63^ in using the mean of all cells to accounting for variation in background staining, while preserving true 0 values and conveying interpretable meaning, (i.e., a CLR value of 1.1 represents ∼2-fold difference in intensity over the mean for the tissue). For high-level cell type assignment, we applied a rule-based classification approach using hand-gated cutoffs of key protein markers according to the following hierarchical criteria.

• Treg: FOXP3 > 1.4 and CD4 > 1.1
• CD8 T cell: CD8 > 1.4 and CD3e > 1.0
• CD4 T cell: CD4 > 1.1, CD3e > 1.1, and CD8 < 1.4
• NK cell: NKG2A > 1.1 and CD3e < 1.1
• Macrophage: Any of CD14, CD163, CD68, or CD16a >1.4, and CD20 < 1.5
• CD4 T cell (fallback): CD4 > 1.1
• B cell: CD20 > 1.5
• Tumor: TP63 > 1.0 or Pan-Cytokeratin >1.0
• Stroma: All other cells not meeting the above criteria

A second round of stricter classification was applied to cells initially typed as CD8, CD4, Treg, B cell, or NK cells given the frequent proximity and double-positivity from segmentation spillover artifacts encountered with these cells.

• Treg: FOXP3 > 1.4
• NK cell: NKG2A > 1.1 and CD3e < 1.1 and CD8 < 1.4
• CD8 T cell: CD8 > 1.4 and CD3e > 0.9 and CD8/4 ratio >1.5
• Also required expression of a second T cell marker (GZMK >1.1, GZMB >1.1, TCF-1 > 0.9, or PD1>0.9), or CD8 > 2
• CD4 T cell: CD4 > 1.1 and CD3e > 0.9 and CD8/4 ratio< 1.5
• Also required expression of a second T cell marker (PD-1 > 0.9, TOX >0.9, or TCF-1 > 0.9)
• Cells not meeting the above criteria were passed back for Macrophage/Tumor/Stroma criteria as in initial categorization.

Cell type classifications were examined for accuracy by visual inspection within QuPath for each of the 8 images. Tumor parenchymal areas were annotated via pixel classifier on the PanCK channel (2 μm/px resolution, smoothing 1, threshold 5). Areas of normal squamous epithelia, secretory glands, and keratin debris (panCK+, without DAPI+ nuclei) were given separate annotations after manual review, and cells otherwise classified as tumor (TP63+/panCK+) in these areas were excluded from tumor-specific analyses.

##### CODEX HLA-DR and neighborhood analysis

Cells were classified as HLA-DR positive if the CLR was greater 0.5, a threshold chosen based on initial Otsu threshold for the whole dataset and adjusted slightly after manual inspection. After cell phenotyping, a neighborhood matrix was created using the spatial_count command from scimap package^57^ (v0.14, method = radius, radius = 60 pixels, corresponding to a 30 μm radius), with each row representing a cell and columns representing the counts of each cell type within a 30 μm radius (based on 0.5 μm/pixel image resolution), and yielded a median of 26 neighboring cell for each cell. For each sample (patient, timepoint), the mean count for each type of neighboring cell was calculated separately for HLA-DR positive and negative malignant cells, and the ratio compared between them. The ratio of neighboring cell types between HLA-DR positive and negative tumor cells was compared by one-tailed Student’s t test.

#### Analysis of BRB-seq data

Reads were then aligned to the mm10 mouse genome (GRCm38) using STARsolo (STAR v2.7.6a),^53^ which simultaneously handled demultiplexing, UMI deduplication, and gene-level quantification by extracting cell barcodes and UMIs from Read 1. Genes with <10 counts in one or more samples were removed. Normalized count matrices were generated using size-factor normalization implemented in pyDESeq2 (v0.4.11) in Python (v3.10.12) within the JupyterLab (v4.0.11) interactive development environment.^54^ All expression data was subsequently integrated into AnnData objects (v 0.10.9). Statistical significance for differential expression was determined using the Wald test, followed by Benjamini-Hochberg correction to control for multiple testing. Bulk RNA-seq gene set scoring was performed using the *scanpy.tl.score_genes()* function (Scanpy v1.10.2) applied to the *.raw* attribute of the AnnData object. All plotting was performed using Seaborn (v0.13.2).^55^

### Statistical analysis

In analysis of human single-cell and bulk RNA seq data, statistical testing between values of continuous variables groups was performed using two-tailed Student’s *t* test. One-sample t-tests were used when comparing means of the same samples at different timepoints. *t* test for correlation was used to test the significance of Pearson correlations between continuous variables. Chi-square test was used to compare categorical variables. When multiple testing was performed, FDR-correction was applied using the Benjamini-Hochberg method.

In mouse experiments, for statistical comparisons between continuous variables, data were first assessed for normality using the Shapiro-Wilk test. Comparisons between normally distributed groups were performed using an unpaired two-tailed Student’s *t* test, whereas comparisons between non-normally distributed groups were performed using the Mann-Whitney *U* test.

In all analyses, asterisks denote significance by *t* test (*p* < 0.05 = ∗, *p* < 0.01 = ∗∗, *p* < 0.001 = ∗∗∗).

### Additional resources

#### Clinical trial information

This study sequenced samples collected from patients in a phase 2 clinical trial (NCT02296684) of neoadjuvant pembrolizumab in patients with locally advanced Stage III/IVB surgically resectable HNSCC.

## Supplemental information

Document S1. Figures S1–S5 and Tables S4 and S5

Table S1. Metaprogram signatures by cell type, related to Figure 1

Table S2. Genes recurrently differentially expressed between matched pre- and post-treatment samples by cell type, related to Figure 2

Table S3. Differentially expressed genes between tumors receiving anti-PD-1 therapy with either anti-IFN-γ neutralizing antibody or IgG control, related to Figure 3

Document S2. Article plus supplemental information
