# Supplementary material for: Single-cell analysis highlights the significance of malignant cell IFN/MHC-II for immunotherapy response in head and neck squamous cell carcinoma
Source: Cell Rep Med. 2026 Mar 31;7(4):102715. doi: 10.1016/j.xcrm.2026.102715 (PMC13130666; doi:10.1016/j.xcrm.2026.102715)
Supplement: Document S1. Figures S1–S5 and Tables S4 and S5 [file mmc1.pdf]

Cell Reports Medicine, Volume *7*

Supplemental information

Single-cell analysis highlights the significance of malignant cell IFN/MHC-II for immunotherapy response in head and neck squamous cell carcinoma

Michael Mints, Reilly A. Sample, Anuraag S. Parikh, Jesse M. Zaretsky, Zongtai Qi, Travis Law, Fudong Wang, Thomas F. Barrett, Riley Mullins, Ashley Reeb, Alissa C. Greenwald, Emily Stoller, Salma Ramadan, Sophie Gerndt, Peter Oppelt, Jessica Ley, Wade Thorstad, Randal C. Paniello, Jason T. Rich, Richard A. Harbison, Paul A. Zolkind, Ryan S. Jackson, Patrik Pipkorn, Douglas R. Adkins, Ravindra Uppaluri, Itay Tirosh, and Sidharth V. Puram

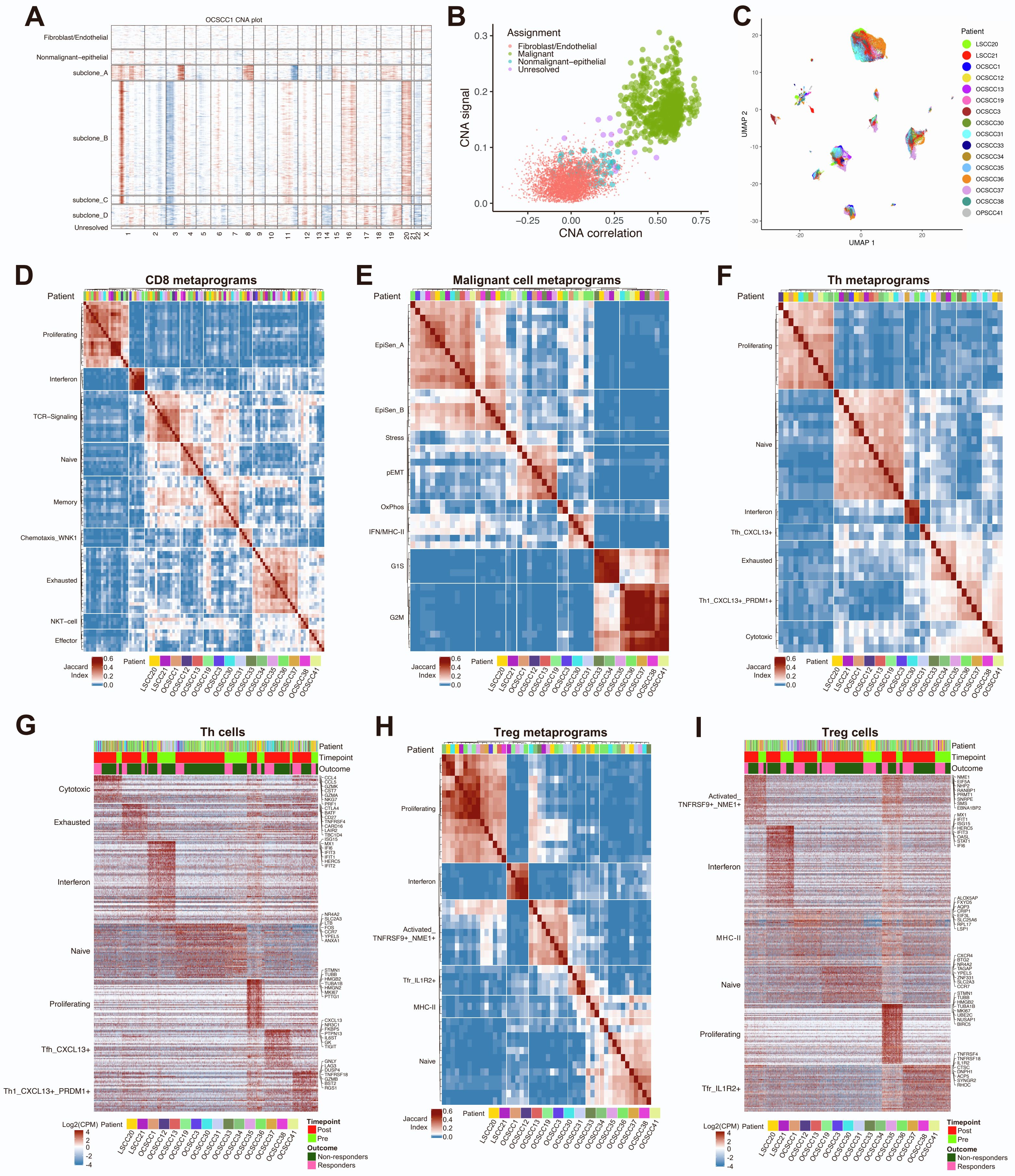
A
OCSCC1 CNA plot
Fibroblast/Endothelial
Nonmalignant–epithelial
subclone_A
subclone_B
subclone_C
subclone_D
Unresolved
1 2 3 4 5 6 7 8 9 10 11 12 13 14 15 16 17 18 19 20 21 22 X
B
Assignment
Fibroblast/Endothelial
Malignant
Nonmalignant–epithelial
Unresolved
CNA signal
CNA correlation
C
Patient
LSCC20
LSCC21
OCSCC1
OCSCC12
OCSCC13
OCSCC19
OCSCC3
OCSCC30
OCSCC31
OCSCC33
OCSCC34
OCSCC35
OCSCC36
OCSCC37
OCSCC38
OPSCC41
UMAP 1
UMAP 2
D
CD8 metaprograms
Patient
Proliferating
Interferon
TCR–Signaling
Naive
Memory
Chemotaxis_WNK1
Exhausted
NKT–cell
Effector
Jaccard Index
E
Malignant cell metaprograms
EpiSen_A
EpiSen_B
Stress
pEMT
OxPhos
IFN/MHC-II
G1S
G2M
F
Th metaprograms
Proliferating
Naive
Interferon
Tfh_CXCL13+
Exhausted
Th1_CXCL13+_PRDM1+
Cytotoxic
G
Th cells
Timepoint
Outcome
Cytotoxic
Exhausted
Interferon
Naive
Proliferating
Tfh_CXCL13+
Th1_CXCL13+_PRDM1+
Log2(CPM)
Post
Pre
Non-responders
Responders
H
Treg metaprograms
Proliferating
Interferon
Activated_ TNFRSF9+_NME1+
Tfr_IL1R2+
MHC-II
Naive
I
Treg cells
Activated_ TNFRSF9+_NME1+
Interferon
MHC-II
Naive
Proliferating
Tfr_IL1R2+

Supplementary Figure 1. Single cell RNA-seq in pre- and post-immunotherapy HNSCC samples. Related to Figure 1.

A) Plot of inferred copy number aberrations (CNA) in epithelial and stromal cells from patient OCSCC1, inferred by taking a 100-gene moving average of relative expression values across the transcriptome and normalizing these values by stromal cells from the same patient (**Methods**). Rows represent cells, arranged by assigned cell type, or genetic subclones in malignant epithelial cells. Columns are genes, arranged by chromosomal position. Unresolved cells are epithelial cells with intermediate CNA signal, not distinctly classified as malignant or nonmalignant.

B) Scatter plot of metrics used to define malignant cells (**Methods**) from patient OCSCC1. Large points are epithelial cells, colored by assignment, small points are stromal reference cells. Y-axis represents CNA signal for each cell – the strength of inferred CNAs across relevant chromosomal regions. X-axis represents CNA correlation – the similarity of each cell's CNA pattern to that of the cells with the strongest CNA signal.

C) UMAP shows 117,012 cells that passed QC and had a confidently assigned cell type colored by patient.

D) Correlation plot shows hierarchical clustering of 94 NMF-derived program signatures from the CD8 T-cells of 16 patients. Signatures are clustered by Jaccard overlap. Groups of signatures, from which meta-programs are derived, are annotated on the left. Top panel shows the patient origin for each program.

E) Correlation plot shows hierarchical clustering of 51 NMF-derived program signatures from the malignant cells of 16 patients. Signatures are clustered by Jaccard overlap. Groups of signatures, from which meta-programs are derived, are annotated on the left. Top panel shows the patient origin for each program.

F) Correlation plot shows Hierarchical clustering of 44 NMF-derived program signatures from the T-helper cells of 15 patients. Signatures are clustered by Jaccard overlap. Groups of signatures,

from which meta-programs are derived, are annotated on the left. Top panel shows the patient origin for each program.

G) Heatmap shows expression of meta-program genes (rows) in all T-helper cells (columns). Cells are annotated by patient of origin, sampling timepoint and outcome.

H) Correlation plot shows hierarchical clustering of 48 NMF-derived program signatures from the T-regulatory cells of 15 patients. Signatures are clustered by Jaccard overlap. Groups of signatures, from which meta-programs are derived, are annotated on the left. Top panel shows the patient origin for each program.

I) Heatmap shows expression of meta-program genes (rows) in all T-regulatory cells (columns). Cells are annotated by patient of origin, sampling timepoint and outcome.

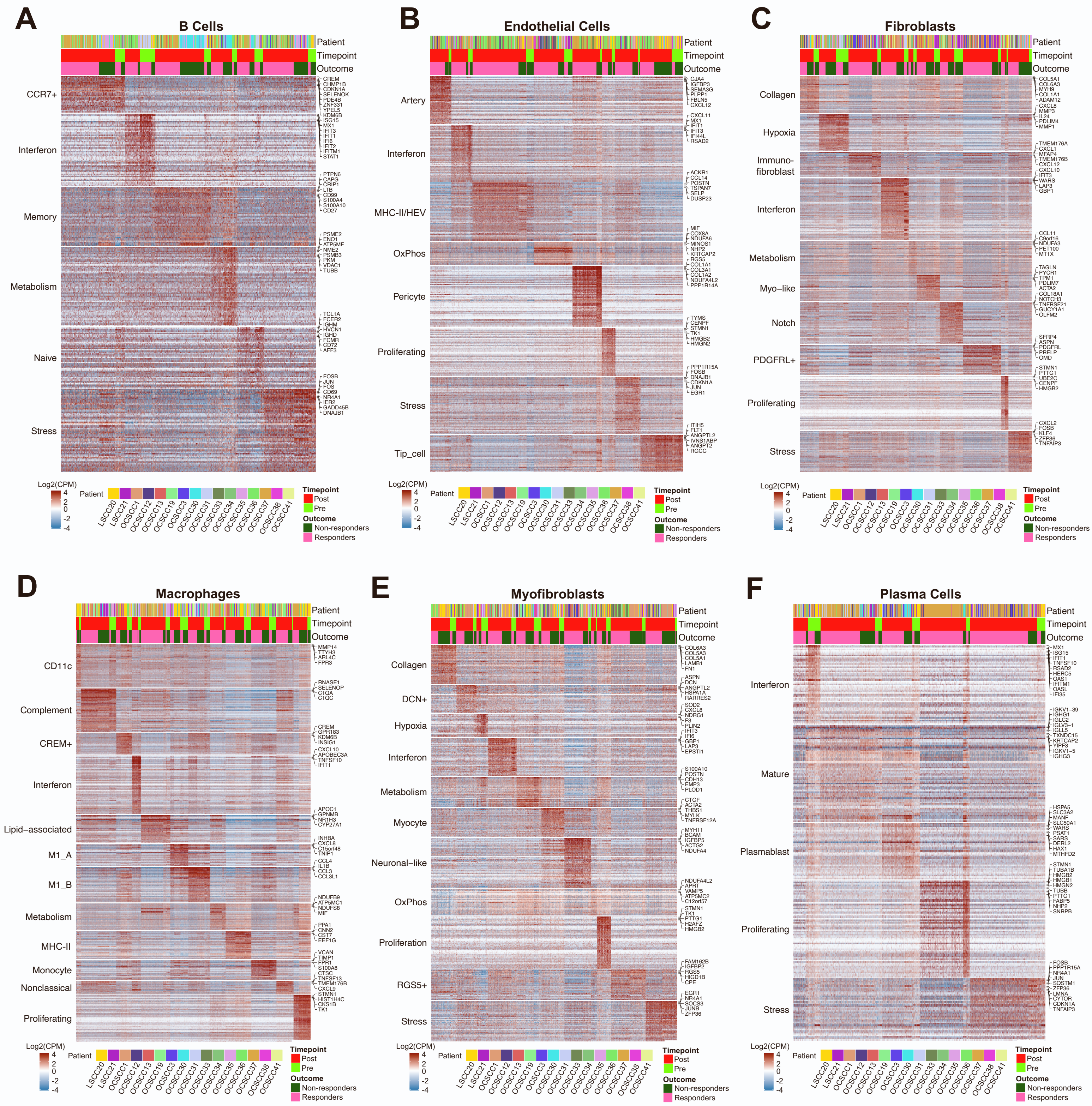
A
B Cells
Patient
Timepoint
Outcome
CCR7+
Interferon
Memory
Metabolism
Naive
Stress
CREM
CHMP1B
CDKN1A
SELENOK
PDE4B
ZNF331
YPEL5
KDM6B
ISG15
MX1
IFIT3
IFIT1
IFI6
IFIT2
IFITM1
STAT1
PTPN6
CAPG
CRIP1
LTB
CD99
S100A4
S100A10
CD27
PSME2
ENO1
ATP5MF
NME2
PSMB3
PKM
VDAC1
TUBB
TCL1A
FCER2
IGHM
HVCN1
IGHD
FCMR
CD72
AFF3
FOSB
JUN
FOS
CD69
NR4A1
IER2
GADD45B
DNAJB1
B
Endothelial Cells
Artery
Interferon
MHC-II/HEV
OxPhos
Pericyte
Proliferating
Stress
Tip_cell
GJA4
IGFBP3
SEMA3G
PLPP1
FBLN5
CXCL12
CXCL11
MX1
IFIT1
IFIT3
IFI44L
RSAD2
ACKR1
CCL14
POSTN
TSPAN7
SELP
DUSP23
MIF
COX8A
NDUFA6
MINOS1
NHP2
KRTCAP2
RGS5
COL1A1
COL3A1
COL1A2
NDUFA4L2
PPP1R14A
TYMS
CENPF
STMN1
TK1
HMGB2
HMGN2
PPP1R15A
FOSB
DNAJB1
CDKN1A
JUN
EGR1
ITIH5
FLT1
ANGPTL2
IVNS1ABP
ANGPT2
RGCC
C
Fibroblasts
Collagen
Hypoxia
Immuno-fibroblast
Interferon
Metabolism
Myo-like
Notch
PDGFRL+
Proliferating
Stress
COL5A1
COL6A3
MYH9
COL1A1
ADAM12
CXCL8
MMP3
IL24
PDLIM4
MMP1
TMEM176A
CXCL1
MFAP4
TMEM176B
CXCL12
CXCL10
IFIT3
WARS
LAP3
GBP1
CCL11
C3orf16
NDUFA3
PET100
MT1X
TAGLN
PYCR1
TPM1
PDLIM7
ACTA2
COL18A1
NOTCH3
TNFRSF21
GUCY1A1
OLFM2
SFRP4
ASPN
PDGFRL
PRELP
OMD
STMN1
PTTG1
UBE2C
CENPF
HMGB2
CXCL2
FOSB
KLF4
ZFP36
TNFAIP3
Log2(CPM)
4
2
0
-2
-4
Patient
LSCC20
LSCC21
OCSCC1
OCSCC12
OCSCC13
OCSCC19
OCSCC3
OCSCC30
OCSCC31
OCSCC33
OCSCC34
OCSCC35
OCSCC36
OCSCC37
OCSCC38
OCSCC41
Timepoint
Post
Pre
Outcome
Non-responders
Responders
D
Macrophages
CD11c
Complement
CREM+
Interferon
Lipid–associated
M1_A
M1_B
Metabolism
MHC-II
Monocyte
Nonclassical
Proliferating
MMP14
TTYH3
ARL4C
FPR3
RNASE1
SELENOP
C1QA
C1QC
CREM
GPR183
KDM6B
INSIG1
CXCL10
APOBEC3A
TNFSF10
IFIT1
APOC1
GPNMB
NR1H3
CYP27A1
INHBA
CXCL8
C15orf48
TNIP1
CCL4
IL1B
CCL3
CCL3L1
NDUFB9
ATP5MC1
NDUFS8
MIF
PPA1
CNN2
CST7
EEF1G
VCAN
TIMP1
FPR1
S100A8
CTSC
TNFSF13
TMEM176B
CXCL9
STMN1
HIST1H4C
CKS1B
TK1
E
Myofibroblasts
Collagen
DCN+
Hypoxia
Interferon
Metabolism
Myocyte
Neuronal–like
OxPhos
Proliferation
RGS5+
Stress
COL6A3
COL5A3
COL5A1
LAMB1
FN1
ASPN
DCN
ANGPTL2
HSPA1A
RARRES2
SOD2
CXCL8
NDRG1
F3
PLIN2
IFIT3
IFI6
GBP1
LAP3
EPSTI1
S100A10
POSTN
CDH13
EMP3
PLOD1
CTGF
ACTA2
THBS1
MYLK
TNFRSF12A
MYH11
BCAM
IGFBP5
ACTG2
NDUFA4
NDUFA4L2
APRT
VAMP5
ATP5MC2
C12orf57
STMN1
TK1
PTTG1
H2AFZ
HMGB2
FAM162B
IGFBP2
RGS5
HIGD1B
CPE
EGR1
NR4A1
SOCS3
JUNB
ZFP36
F
Plasma Cells
Interferon
Mature
Plasmablast
Proliferating
Stress
MX1
ISG15
IFIT1
TNFSF10
RSAD2
HERC5
OAS1
IFITM1
OASL
IFI35
IGKV1–39
IGHG1
IGLC2
IGLV3–1
IGLL5
TXNDC15
KRTCAP2
YIPF3
IGKV1–5
IGHG3
HSPA5
SLC3A2
MANF
SLC50A1
WARS
PSAT1
SARS
DERL2
HAX1
MTHFD2
STMN1
TUBA1B
HMGB2
HMGB1
HMGN2
TUBB
PTTG1
FABP5
NHP2
SNRPB
FOSB
PPP1R15A
NR4A1
JUN
SQSTM1
ZFP36
LMNA
CYTOR
CDKN1A
TNFAIP3

Supplementary Figure 2. Single cell RNA-seq in pre- and post-immunotherapy HNSCC samples. Related to Figure 1.

Heatmaps show expression of meta-program genes (rows) in all B cells (**A,** columns), endothelial cells (**B**), fibroblasts (**C**), macrophages (**D**), myofibroblasts (**E**), and plasma cells (**F**). Cells are annotated by patient of origin, sampling timepoint and outcome.

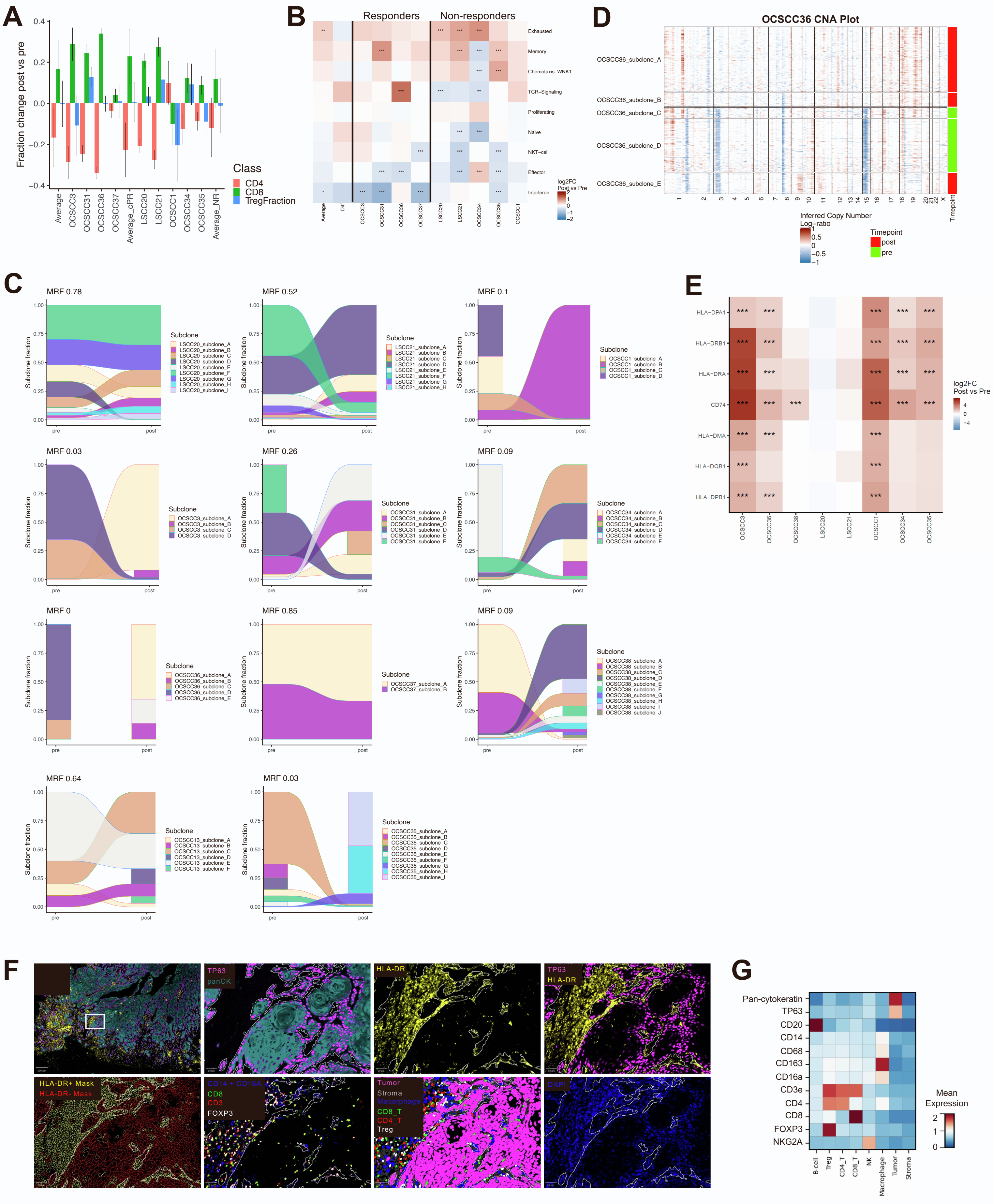
A
Fraction change post vs pre
Average
OCSCC3
OCSCC31
OCSCC36
OCSCC37
Average_cPR
LSCC20
LSCC21
OCSCC1
OCSCC34
OCSCC35
Average_NR
Class
CD4
CD8
TregFraction
B
Responders
Non-responders
Exhausted
Memory
Chemotaxis_WNK1
TCR-Signaling
Proliferating
Naive
NKT-cell
Effector
Interferon
Average
Diff
log2FC
Post vs Pre
D
OCSCC36 CNA Plot
OCSCC36_subclone_A
OCSCC36_subclone_B
OCSCC36_subclone_C
OCSCC36_subclone_D
OCSCC36_subclone_E
Timepoint
Inferred Copy Number
Log-ratio
post
pre
C
MRF 0.78
MRF 0.52
MRF 0.1
MRF 0.03
MRF 0.26
MRF 0.09
MRF 0
MRF 0.85
MRF 0.09
MRF 0.64
MRF 0.03
Subclone fraction
Subclone
pre
post
E
HLA-DPA1
HLA-DRB1
HLA-DRA
CD74
HLA-DMA
HLA-DQB1
HLA-DPB1
OCSCC3
OCSCC36
OCSCC38
LSCC20
LSCC21
OCSCC1
OCSCC34
OCSCC35
log2FC
Post vs Pre
F
TP63
panCK
HLA-DR
TP63
HLA-DR
HLA-DR+ Mask
HLA-DR- Mask
CD14 + CD16A
CD8
CD3
FOXP3
Tumor
Stroma
Macrophage
CD8_T
CD4_T
Treg
DAPI
G
Pan-cytokeratin
TP63
CD20
CD14
CD68
CD163
CD16a
CD3e
CD4
CD8
FOXP3
NKG2A
B-cell
Treg
CD4_T
CD8_T
NK
Macrophage
Tumor
Stroma
Mean
Expression

Supplementary Figure 3. T cell and malignant cell subsets are altered following anti-PD1 treatment. highlighted by post-treatment upregulation of malignant cell MHC-II and interferon response genes. Related to Figures 2 and 3.

A) Bar plot shows the change in T cell composition for 9 patients with at least 100 T cells with a confidently assigned subtype both before and after treatment. For each patient, 20% of the total number of T cells was resampled 100 times. The Y-axis shows average change in the fraction of each T cell subset across 100 resampling runs. Error bars denote standard error. CD4 includes all CD4+ T cells, while TregFraction shows the fraction of CD4+ cells classified as Tregs. Averages for all patients, responders and non-responders were derived by averaging the patient means.

B) Heatmap shows the differences in CD8 metaprogram scores for each single patient with at least 50 cells at each timepoint. The average is derived by averaging all mean values, the Diff column is the difference between responder and non-responder means. Asterisks denote significance by t-test ($p<0.05$=*, $p<0.01$=**, $p<0.0001$=***). Only fold changes above 1.3 were marked as significant.

C) For each patient with paired pre- and posttreatment samples, plots show how the distribution of malignant cells across genetic subclones changes between sampling timepoints. MRF=maximum retained fraction, or the maximal fraction of malignant cells that theoretically belong to the same subclone before and after treatment (see **Methods**).

D) Plot of inferred copy number aberrations (CNA) in epithelial cells from patient OCSCC36, inferred through taking a 100-gene moving average of relative expression values across the transcriptome (**Methods**). Rows represent cells, arranged by genetic subclones, and columns genes, arranged by chromosomal position. Rightmost column shows sampling timepoint.

E) Heatmap shows change in malignant cell MHC-II genes from the malignant-IFN/MHC-II program post-treatment, relative to pre-treatment. Y-axis shows MHC-II genes from the malignant-IFN/MHC-II program, and X-axis shows all samples with matched pre- and post-

treatment data from malignant cells. Color shows log2 fold change post- versus pretreatment. ***=p<0.0001 (FDR-corrected) and abs(log2FC)>1.

F) Post-treatment multiplex immunofluorescence (mIF) Phenocycler images of FFPE stained tissues show representative areas of HLA-DR negative malignant cells despite high adjacent stromal expression from a representative tumor (OCSCC35). Top left image shows whole slide view, while other panels represent magnified fields of view from inset, showing (from top left to bottom right) malignant cells highlighted by TP63 and panCK, HLA-DR expression, HLA-DR expression within malignant cells, assigned cell boundaries following Instanseg cell segmentation and HLA-DR +/- classification based on centered log-ratio threshold, staining for T-cell and macrophages (CD14 and CD16A combined in blue, CD8 green, CD3 red, FOXP3 white), post-classification cell-type assignment, and DNA stain for all cells.

G) Heatmap shows post-classification mean expression levels for markers used for cell typing.

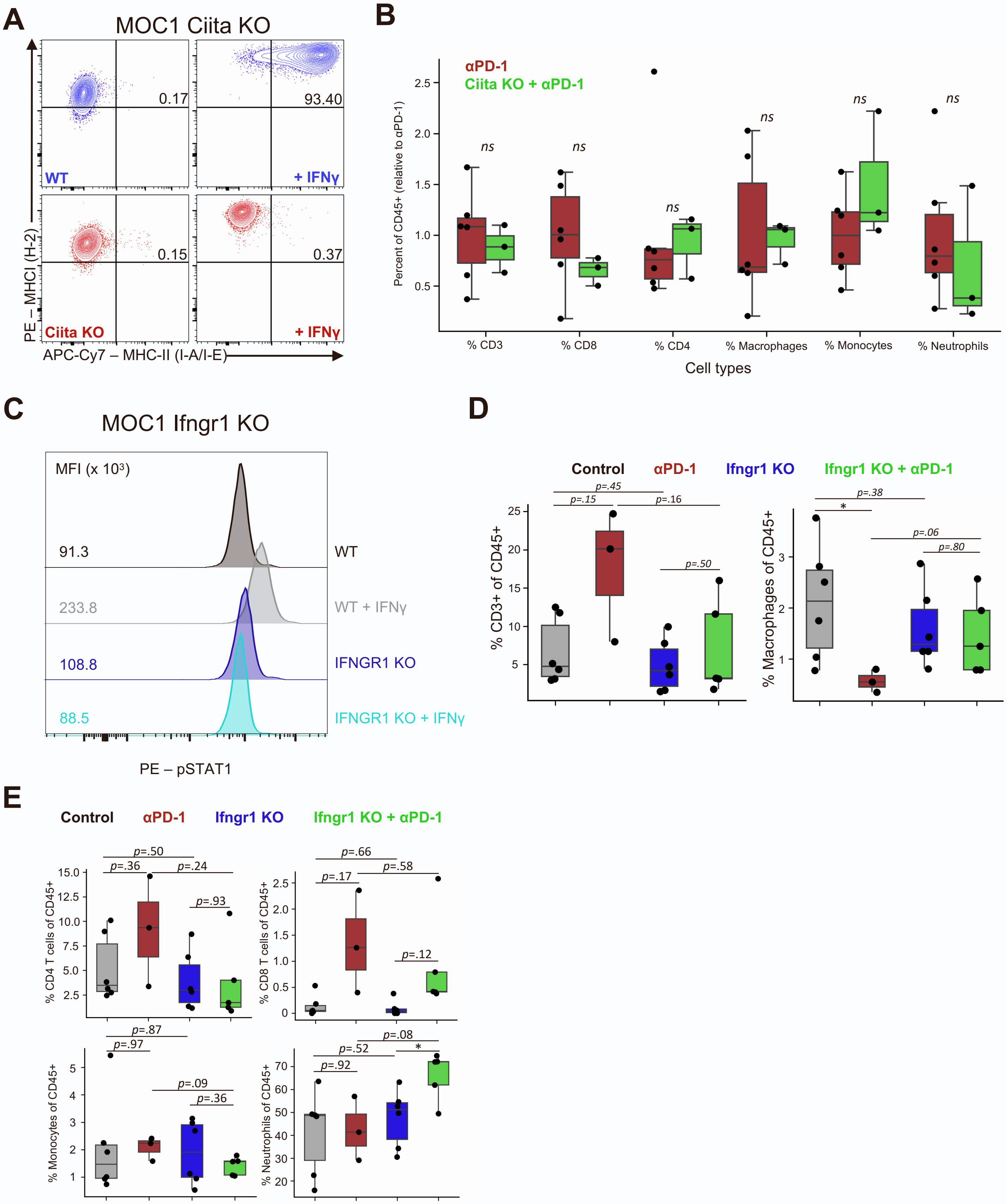
A
MOC1 Ciita KO
0.17
93.40
WT
+ IFNγ
0.15
0.37
Ciita KO
+ IFNγ
PE – MHCI (H-2)
APC-Cy7 – MHC-II (I-A/I-E)
B
αPD-1
Ciita KO + αPD-1
Percent of CD45+ (relative to αPD-1)
ns
% CD3
% CD8
% CD4
% Macrophages
% Monocytes
% Neutrophils
Cell types
C
MOC1 Ifngr1 KO
MFI (x 10³)
91.3
WT
233.8
WT + IFNγ
108.8
IFNGR1 KO
88.5
IFNGR1 KO + IFNγ
PE – pSTAT1
D
Control
αPD-1
Ifngr1 KO
Ifngr1 KO + αPD-1
p=.45
p=.15
p=.16
p=.50
% CD3+ of CD45+
p=.38
*
p=.06
p=.80
% Macrophages of CD45+
E
Control
αPD-1
Ifngr1 KO
Ifngr1 KO + αPD-1
p=.50
p=.36
p=.24
p=.93
% CD4 T cells of CD45+
p=.66
p=.17
p=.58
p=.12
% CD8 T cells of CD45+
p=.87
p=.97
p=.09
p=.36
% Monocytes of CD45+
p=.08
p=.52
*
p=.92
% Neutrophils of CD45+

Supplementary Figure 4. Ciita and Ifngr1 knockouts in MOC1 cells and MOC1 derived tumors. Related to Figures 3 and 4.

A) Representative flow cytometry plots show WT or *Ciita* KO MOC1 cell lines. Following *Ciita* KO or control nucleofection, cell lines underwent negative selection for MHC-II expression by FACS. Plots here show selected cell lines upon 48 hours of re-exposure to 100 ng/mL IFN-γ or vehicle control.

B) Box plots show infiltrating cell type proportions between WT or *Ciita* KO tumors treated with anti-PD-1. Values for each comparison were scaled by the average proportion of WT tumor-bearing mice treated with anti-PD-1. Each dot represents one mouse. Asterisks ($p<0.05$=*, $p<0.01$=**, $p<0.001$=***) denote significance by t-test.

C) Representative flow cytometry plots show WT or Ifngr1 KO MOC1 cell lines. Following Ifngr1 KO or control nucleofection, cell lines underwent negative selection for PD-L1 expression by FACS. Plots here show selected cell lines upon 6 hours of re-exposure to 100 ng/mL IFN-γ or vehicle control.

D) Box plots show infiltrating T cell and macrophage proportions between WT or *Ciita* KO tumors treated with anti-PD-1 or IgG control. T cells were gated live cells/$CD45^+$/$CD3^+$. Macrophages were gated live cells/$CD45^+$/$CD3^-$/$CD11b^+$/Ly-$6G^-$/Ly-$6C^-$/F4/80^+. Each dot represents one mouse. Asterisks ($p<0.05$=*, $p<0.01$=**, $p<0.001$=***) denote significance by t-test.

E) Box plots show infiltrating cell type proportions between WT or *Ciita* KO tumors treated with anti-PD-1 or IgG control. CD4 and CD8 T cells were gated live cells/$CD45^+$/$CD3^+$/$CD4^+$ or $CD8^+$. Monocytes were gated live cells/$CD45^+$/$CD3^-$/$CD11b^+$/Ly-$6C^+$/Ly-$6G^-$. Neutrophils were gated live cells/$CD45^+$/$CD3^-$/$CD11b^+$/Ly-$6G^+$/Ly-$6C^{int}$. Each dot represents one mouse. Asterisks ($p<0.05$=*, $p<0.01$=**, $p<0.001$=***) denote significance by t-test.

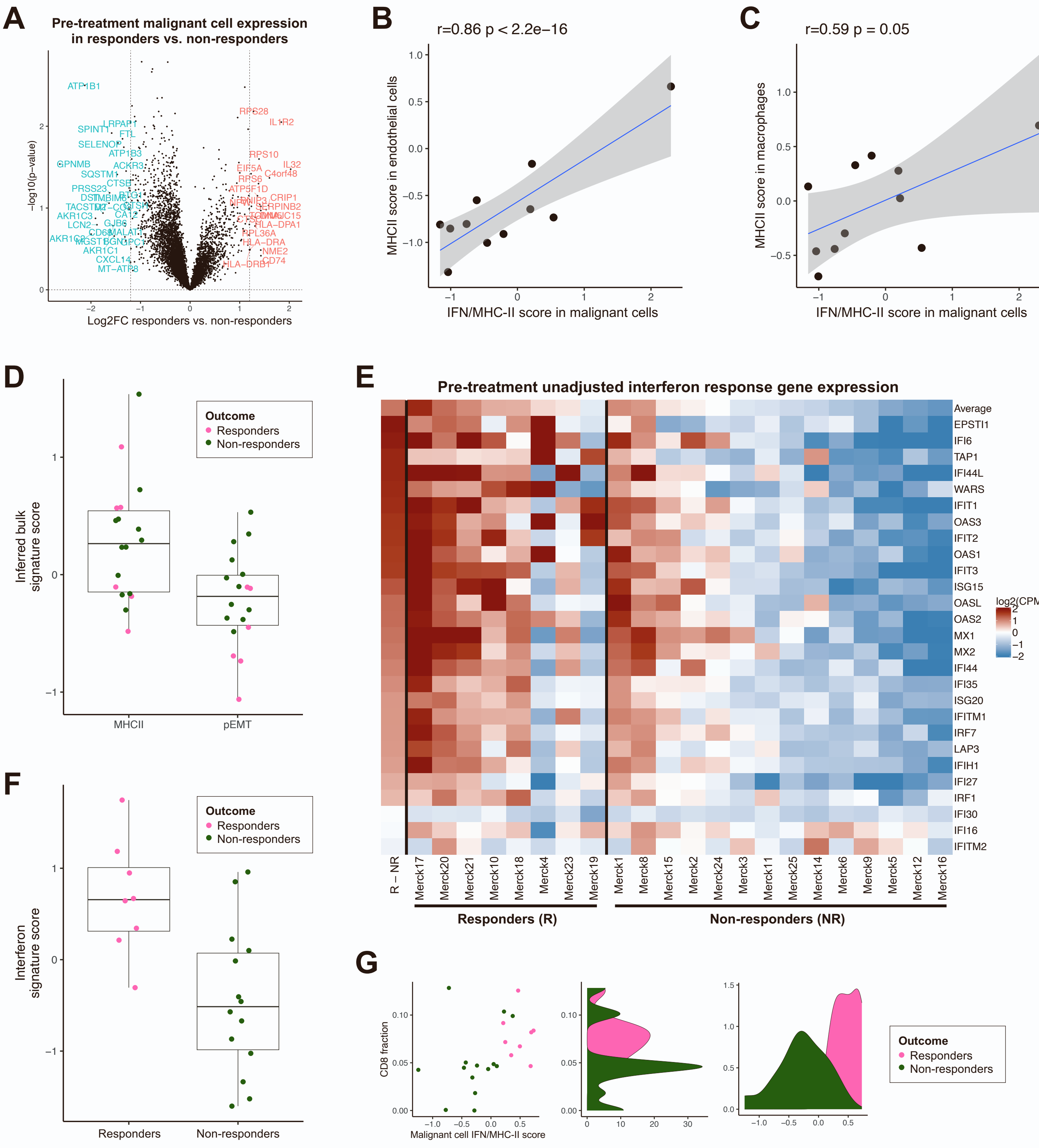
A
Pre-treatment malignant cell expression in responders vs. non-responders
−log10(p−value)
Log2FC responders vs. non-responders
ATP1B1
SPINT1
SELENOP
GPNMB
SQSTM1
PRSS23
AKR1C3
LCN2
AKR1C2
AKR1C1
CXCL14
MT−ATP8
RPS28
IL1R2
RPS10
IL32
EIF5A
C4orf48
RPS6
ATP5F1D
CRIP1
SERPINB2
HLA−DPA1
RPL36A
HLA−DRA
NME2
CD74
HLA−DRB1
B
r=0.86 p < 2.2e−16
MHCII score in endothelial cells
IFN/MHC-II score in malignant cells
C
r=0.59 p = 0.05
MHCII score in macrophages
IFN/MHC-II score in malignant cells
D
Inferred bulk signature score
MHCII
pEMT
Outcome
Responders
Non-responders
E
Pre-treatment unadjusted interferon response gene expression
Average
EPSTI1
IFI6
TAP1
IFI44L
WARS
IFIT1
OAS3
IFIT2
OAS1
IFIT3
ISG15
OASL
OAS2
MX1
MX2
IFI44
IFI35
ISG20
IFITM1
IRF7
LAP3
IFIH1
IFI27
IRF1
IFI30
IFI16
IFITM2
log2(CPM)
R − NR
Merck17
Merck20
Merck21
Merck10
Merck18
Merck4
Merck23
Merck19
Merck1
Merck8
Merck15
Merck2
Merck24
Merck3
Merck11
Merck25
Merck14
Merck6
Merck9
Merck5
Merck12
Merck16
Responders (R)
Non-responders (NR)
F
Interferon signature score
Responders
Non-responders
Outcome
Responders
Non-responders
G
CD8 fraction
Malignant cell IFN/MHC-II score
Outcome
Responders
Non-responders

Supplementary Figure 5. MHC-II upregulation in malignant cells correlates with response to anti-PD1 treatment. Related to Figure 5.

A) Volcano plot shows the genes with largest expression differences between responders and non-responders among malignant cells in the pre-treatment samples. Y-axis denotes -log10(p-value) by t-test.

B-C) Scatter plots show, for each pre-treatment sample, the average expression of the immune metaprogram score in malignant cells (x-axis) and of the MHC-II/HEV metaprogram score in endothelial cells (**B**), and MHC-II metaprogram score in macrophages (**C**), respectively (y-axis). Pearson correlation and p-value by t-test for correlation are shown.

D) Box plot shows, for each of 18 matched bulk samples (points colored by outcome) the average post-treatment change in gene expression (y-axis) of MHC-II (adjusted by macrophage and DC fractions) and pEMT (adjusted by fibroblast and myofibroblast fractions) genes, respectively.

E) Heatmap shows, for all 22 pre-treatment bulk samples, the unadjusted expression of interferon response genes.

F) Box plot shows average expression of the interferon gene expression signature, adjusted for abundance of T-cells and macrophages, for all 22 pre-treatment samples (points), grouped by outcome (x-axis).

G) (Left) Plot shows, on the x-axis, the deconvoluted cancer-specific IFN/MHC-II signature score and inferred CD8 fractions on the y-axis. (Middle/Right) Density plots show distribution of values across the axes for responders and non-responders.

Supplementary Table S4. Multivariate Cox regression model of inferred cell type fractions and signature scores in pre-treatment bulk samples predictive of outcome. Related to Figure 5.

	Estimate	Std. Error	t value	Pr(>|t|)
(Intercept)	0.53075049	0.57825043	0.91785576	0.37321755
CD8 fraction	-0.6659427	3.96156172	-0.1681011	0.86874935
T-helper fraction	-1.1744814	2.21640522	-0.5299037	0.6039345
Malignant IFN/MHC-II score	0.55243639	0.24162881	2.28630188	0.03719535
Macrophage fraction	-0.9873722	4.7288935	-0.2087956	0.83741672
Interferon score	0.12692946	0.17528865	0.7241168	0.48013637
Treg fraction	0.79553453	4.15213448	0.19159652	0.85062773

Supplementary Table S5. Recurrently differentially expressed genes between transcriptionally NK-like cells with and without T-cell receptor from the same patient. Related to STAR Methods.

Gene	No. Patients	Fraction. Patients	logFC	Group
CD3D	11	1	4.69141473	TCR present
CD3E	8	0.72727273	2.9094538	TCR present
CD8A	6	0.54545455	4.02015697	TCR present
CD3G	5	0.45454546	3.06557292	TCR present
S100A4	5	0.45454546	2.78972208	TCR present
IL32	5	0.45454546	4.05269076	TCR present
TYROBP	8	0.72727273	5.02290001	TCR absent
FCER1G	8	0.72727273	4.30467587	TCR absent
TRDC	8	0.72727273	2.23161069	TCR absent
SH2D1B	7	0.63636364	1.7950344	TCR absent
GNLY	5	0.45454546	4.93549736	TCR absent
KLRC1	5	0.45454546	2.72978795	TCR absent
KRT86	5	0.45454546	1.74588568	TCR absent
LAT2	5	0.45454546	1.61363956	TCR absent
